# Gastrointestinal pH and Transit Time Profiling in Healthy Volunteers Using the IntelliCap System Confirms Ileo-Colonic Release of ColoPulse Tablets

**DOI:** 10.1371/journal.pone.0129076

**Published:** 2015-07-15

**Authors:** Jacoba M. Maurer, Reinout C. A. Schellekens, Hèlen M. van Rieke, Christoph Wanke, Ventzeslav Iordanov, Frans Stellaard, Klaus D. Wutzke, Gerard Dijkstra, Margot van der Zee, Herman J. Woerdenbag, Henderik W. Frijlink, Jos G. W. Kosterink

**Affiliations:** 1 University of Groningen, University Medical Center Groningen, Department of Clinical Pharmacy and Pharmacology, Groningen, The Netherlands; 2 Medimetrics Personalized Drug Delivery BV, Eindhoven, The Netherlands; 3 University of Groningen, University Medical Center Groningen, Department of Laboratory Medicine, Groningen, The Netherlands; 4 University of Rostock, Children’s Hospital, Research Laboratory Gastroenterology and Nutrition, Rostock, Germany; 5 University of Groningen, University Medical Center Groningen, Department of Gastroenterology and Hepatology, Groningen, The Netherlands; 6 University of Groningen, Department Pharmacy, Section of Pharmaceutical Technology and Biopharmacy, Groningen, The Netherlands; 7 University of Groningen, Department Pharmacy, Section of Pharmacotherapy and Pharmaceutical Care, Groningen, The Netherlands; The Lee Kong Chian School of Medicine, SINGAPORE

## Abstract

**Introduction:**

ColoPulse tablets are an innovative development in the field of oral dosage forms characterized by a distal ileum and colon-specific release. Previous studies in humans showed release in the ileo-colonic region, but the relationship between gastrointestinal pH and release was not experimentally proven *in vivo*. This information will complete the *in vivo* release-profile of ColoPulse tablets.

**Materials and Methods:**

Release from ColoPulse tablets was studied in 16 healthy volunteers using the dual label isotope strategy. To determine gastrointestinal pH profiles and transit times the IntelliCap system was used. A ColoPulse tablet containing ^13^C-urea and an uncoated, immediate release tablet containing ^15^N_2_-urea were taken simultaneously followed by a standardized breakfast after three hours. Five minutes after intake of the tablets the IntelliCap capsule was swallowed and pH was measured until excretion in the feces. Breath and urine samples were collected for isotope analysis.

**Results:**

Full analysis could be performed in 12 subjects. Median bioavailability of ^13^C -urea was 82% (95% CI 74–94%, range 61–114%). The median lag time (5% release of ^13^C) was 5:42 h (95% CI 5:18–6:18 h, range 2:36–6:36 h,) There was no statistically significant difference between lag time based on isotope signal and colon arrival time (CAT) based on pH (median 5:42 vs 5:31 h p = 0.903). In all subjects an intestinal pH value of 7.0 was reached before release of ^13^C from the ColoPulse tablet occurred.

**Discussion and Conclusions:**

From the combined data from the IntelliCap system and the ^13^C -isotope signal it can be concluded that release from a ColoPulse tablet *in vivo* is not related to transit times but occurs in the ileo-colonic region after pH 7.0 is reached. This supports our earlier findings and confirms that the ColoPulse system is a promising delivery system for targeting the distal ileum and colon.

**Trial Registration:**

ISRCTN Registry 18301880

## Introduction

Distal ileum and Colon-specific delivery of medicines is clinically relevant because their efficacy can be improved, side effects can be reduced and the bioavailability of drugs that are metabolized or poorly absorbed in the higher parts of the small intestine can be enhanced. This offers interesting perspectives for the treatment of for instance inflammatory bowel diseases with peptides [1].

In the literature different strategies for colon-specific delivery have been described. They include pH-responsive systems, time-based systems and systems triggered by the colon flora, as well as combinations of such systems [1,2]. The recently developed ColoPulse technology is a promising pH-responsive system being able to specifically deliver the active substance to the ileo-colonic region. Because of the non-percolating incorporation of a super-disintegrant in the coating, by which a more reliable and pulsatile release is achieved, it differs from other pH-responsive systems. The dissolution of this coating is triggered by the physiologically occurring increase in pH from 5.5 in the upper small intestine to 7.5 in the ileo-colonic region [3].

Until now bioavailability from ColoPulse dosage forms has been studied in healthy volunteers and in patients with Crohn’s disease using stable isotopes of urea [4,5,6]. All studies showed a mean total bioavailability of ^13^C-urea of > 76%, but the relationship between gastrointestinal pH and release from a ColoPulse dosage form has so far not been proven experimentally *in vivo*. To obtain more insight in the behaviour and functioning of ColoPulse tablets and capsules *in vivo*, studies on the gastrointestinal pH with the concomitant administration of a ColoPulse tablet are justified.

In the literature different devices for gastrointestinal pH measurement have been described [7]. They can be divided in two subgroups: the “static” devices and the “freely moving” devices. With representatives of the first group only the oesophageal, intra-gastric or peri-mucosal colonic pH can be measured. Their main disadvantage is the inability to measure the pH along the entire length of the gastrointestinal tract. The second group mainly comprises pH sensitive wireless radiotelemetry capsules (RTC). After administration of a RTC the subject is able to perform normal daily activities. Emitted radio signals are detected by a recorder mostly worn around the waist. If the device functions properly, measuring ends when the RTC is excreted with the feces. Disadvantages of the RTCs used so far are the frequent loss of signals, batteries running out of power before excretion, large pH-drift (up to approximately 1 unit) and difficulties in determining the exact location of the capsule in the gastrointestinal tract. In Table 1 a summary of the available literature on freely moving devices studied in inflammatory bowel diseases and / or healthy volunteers is presented [8–21].

**Table 1:** Summary of available literature on gastrointestinal pH measurement with freely moving devices in healthy volunteers and / or inflammatory bowel diseases.

Study	Device	Subjects	Battery life	pH Sampling interval	Position detection	Food intake during study	Data loss	pH drift device during study	Total transit time	Remarks
Watson et al, 1972 [8]	Radiotelemetry Capsule	2 healthy subjects 7 patients with miscellaneous gastrointestinal disorders	10 days	60 min	Abdominal x-ray	Device intake after breakfast. No restrictions in food and beverages	-^a^	0.1 unit	-	
Evans et al, 1988 [9]	Radiotelemetry capsule (Remote control systems Ltd, UK)	72 healthy subjects	-	12 seconds	“locator” to detect highest signal intensity	Device intake after overnight fasting. breakfast after leaving the stomach. No restrictions in food and beverages	In 14 subjects > 75% loss in the small intestine	pH 4: < 0.6 unit pH 9.2 < 1.0 unit	Mean: 23.3 h	Measurement up to 48 h. Median signal loss 20.4%. 2 subjects > 1.0 unit pH drift
Fallingborg et al, 1989 [10]	Radiotelemetry Capsule (Remote control systems Ltd, UK)	39 healthy subjects	-	15–120 min (not between 11 pm and 8 am)	Fluoroscopy	Device intake after overnight fasting; breakfast after leaving the stomach. Food and beverages according to the protocol	-	< 0.9 unit	9–129 h	
Raimundo A et al 1992 [11]	Radiotelemetry Capsule	7 patients with acute colitis 6 patients with ulcerative colitis in remission	-	-	Based on pH	-	-	-	-	
Fallingborg et al, 1993 [12]	Radiotelemetry Capsule	7 patients with ulcerative colitis	-	30 min (not between 11 pm and 8 am)	Fluoroscopy	Device intake after overnight fasting; breakfast after leaving the stomach. No restrictions in food and beverages	-	< 0.4 unit	8 - > 123 h	Measurement max 39h.
Sasaki et al, 1997 [13]	Radiotelemetry Capsule (Remote control systems Ltd, UK)	4 healthy subjects 4 patients with Crohn’s disease	1	1 second	Based on pH, x-ray, contrast colonogram and a radio-directional probe	Device intake after overnight fasting; breakfast after leaving the stomach. Food according to the protocol	-	< 0.5unit	-	
Press et al, 1998 [14]	Radiotelemetry Capsule (7036, Oakfield instruments, UK)	12 healthy subjects 11 patients with ulcerative colitis 15 patients with Crohn’s disease	-	-	“locator” to detect highest signal intensity	Device intake after overnight fasting; breakfast after leaving the stomach. No restrictions in food and beverages	In 4 subjects > 75% loss in 24h	< 0.5 unit	-	Measurement in the colon was marked as unpredictable. 4 subjects had to repeat the study
Fallingborg et al, 1998 [15]	Radiotelemetry Capsule (remote control systems Ltd, UK)	13 healthy subjects 9 patients with Crohn’s disease	-	10–15 min	Fluoroscopy	Device intake after > 8h fasting; breakfast after leaving the stomach	-	<0.4 unit	-	Difference in small intestine transit time between resected patients and healthy volunteers
Ewe et al, 1999 [16]	Radiotelemetry Capsule (7036, Oakfield instruments, UK)	15 healthy subjects 15 patients with Crohn’s Disease 5 patients with ulcerative colitis	24 h	6 seconds	Metal detector	Device intake after > 8h fasting; breakfast after leaving the stomach	6 subjects excluded, several reasons	-	Median 24–31 h	In 1 subject > 2 weeks retention of RTC
Maqbool et al, 2009 [17]	SmartPill, (Buffalo, NY, USA)	10 healthy subjects	5 days	5 seconds, after 24 h 20 seconds	Based on pH	2000 kcal diet, 30% fat. Device intake after breakfast	-	-	-	
Rubin et al, 2009 [18]	Smartpill, (Buffalo, NY, USA)	10 patients with active ulcerative colitis	-	-	Based on pH, motility and temperature	-	-	-	Median 24.6 h	No complications with the device
Lalezari et al, 2012 [19]	SmartPill, (Buffalo, NY, USA)	10 healthy subjects 9 patients with IBS	5 days	5 seconds	Based on pH	Device intake after > 8h fasting; breakfast after leaving the stomach	-	-	-	
Schaar et al, 2013 [20] / Koziolek et al, 2014 [21]	(IntelliCap MedimetricsEindhoven, NL)	2x 10 healthy volunteers	> 48 h	10 seconds	Study 1: based on pH and temperature. Study 2: also based on ^99M^Tc	Device intake with water after overnight fasting. Food 4 h after device intake	Mean 3.5% (one subject 13%)	-	Average 30:34 h	Two publications, same studies

^a^- = no information in publication.

Recently a medical device for the *in vivo* measurement of pH and temperature in the gastrointestinal tract was developed by Medimetrics (Eindhoven, The Netherlands): the IntelliCap system [20,21]. Furthermore the system can be used for electronically controlled drug delivery in defined sections of the gastrointestinal tract to quantify regional drug absorption [22]. It differs from the so far available freely moving RTCs by more accurate and more frequent measurements, minimal signal loss, a built-in drug reservoir and improved battery power (> 72 h) to ensure reliable and complete data acquisition. By combining diagnostic functionalities and the capability to generate adjustable controlled drug release profiles, the IntelliCap system can play a promising role in pharmaceutical drug profiling and formulation development.

In this paper we describe a study performed in healthy volunteers in which we studied the relationship between gastrointestinal pH obtained with the IntelliCap system as well as the release from a ColoPulse tablet to prove that release from a ColoPulse tablet indeed does occur in the ileo-colonic region and after a pH value of ≥ 7.0 is reached.

## Materials and Methods

### Subjects

Sixteen healthy volunteers (10 male, 6 female, age 18–65) were initially included in this study (Table 2). Participant recruitment started January 2011 and ended April 2011. Written informed consent was obtained from all participants. They had no history of gastrointestinal diseases or gastrointestinal surgery. None of the subjects used antibiotics or drugs influencing the gastrointestinal transit time for at least three months prior to the start of the study. A possible *Helicobacter pylori* infection was excluded with a ^13^C-urea breath test (INFAI, Köln, Germany).

**Table 2:** Demographics of included subjects (healthy volunteers).

	Median (range)
**Sex (male / female)**	10 / 6
**Age (year)**	27.5 (19–63)
**Weight (kg)**	77.0 (54.5–121.4)

**Fig 1:**
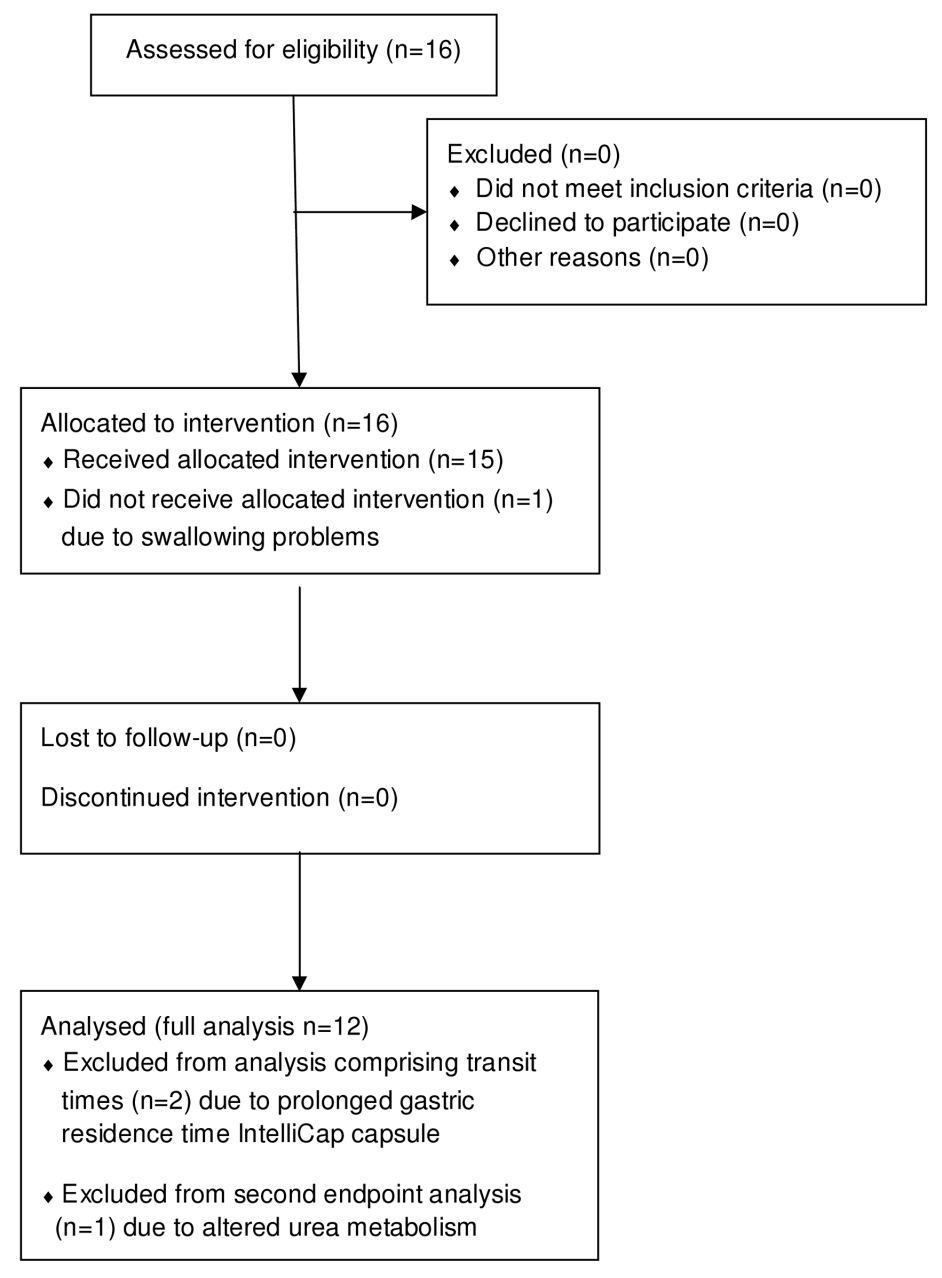
Flowchart.

A flowchart summarizing recruitment and analysis is shown in Fig 1.

### Study-design

This bioavailability study was performed as an open-label, non-randomized, single arm clinical trial and was part of a study described by Maurer et al [6]. The design was based on a previous feasibility study in which two stable isotopes of urea are administered simultaneously [23]. Subjects were fasted from 8 p.m. the day before the test day. Only water, apple juice (until 11 p.m.) and unsweetened tea without sugar were allowed. On the test day an uncoated tablet containing 50 mg ^15^N_2_-urea and a ColoPulse tablet containing 50 mg ^13^C-urea were taken simultaneously at around 8 a.m. with 100 mL apple juice. Five minutes thereafter the IntelliCap capsule was swallowed with another 100 mL apple juice. A standardized breakfast was taken three hours after the intake of the tablets. The meal consisted of a standardized double sandwich and 200 mL unsweetened tea. Approximately 6 and 10 hours after tablet intake, respectively lunch and dinner were taken. There were no food-restrictions for lunch and dinner except foods rich in ^13^C, like corn products, cane sugar and pineapple. During the test day, (that ended at 8 a.m. the next morning) water, apple juice and tea were the only beverages allowed.

Sampling, administration of the tablets and IntelliCap capsule took place in a controlled facility until 5 p.m. Thereafter subjects went home were they continued sampling of breath and urine according to the study protocol. All necessary information was recorded in a diary. A summary of the study design is shown in Table 3.

**Table 3:** Study schedule, activities are marked with an X (T0 is 8 a.m.).

Time (h)	-12	0	1	2	3	4	5	6	7	8	9	10	11	12	13	14	15	24
**Arrival, start fasting**	X																	
**Intake tablets**		X																
**Intake IntelliCap**		X																
**Meal**					X			X				X						
**Urine Sample**		X				X			X		X			X			X	X
**Breath sample**		X	X	X	X	XX	XX	X	XX	XX	XX	XX	XX	X	X	X	X	
**Return home**											X							

### Ethics statement

The study was approved by the ethical committee of the University Medical Center Groningen (ref 2009.188 / EudraCT 2009–01347121) and was performed according to the principles of the Declaration of Helsinki. The study has been registered in the ISRCTN register (ISRCTN18301880). This was not done before the start of recruitment because at that time this was not required by internal procedures and the ethical committee that approved this study.

### IntelliCap system

The IntelliCap system was supplied by Medimetrics (Eindhoven, The Netherlands) and consisted of a capsule and a portable unit. The size of the IntelliCap capsule was 27 x 11 mm. A complete description including illustrations of the IntelliCap system can be found in the literature [20]. The drug reservoir was filled with normal saline solution which had no function in this study and was not expelled from the IntelliCap capsule during the experiments.

Data were measured until excretion of the IntelliCap capsule or until the battery ran out of power. The excretion of the IntelliCap capsule from the body had to be confirmed by collecting the device from the stool. If the IntelliCap capsule was not retrieved within 96 hours it was probably missed and its absence from the body was confirmed with an abdominal X-ray. No follow up was required after retrieving the IntelliCap capsule or confirmation of its absence by X-ray.

### Analysis of pH profiles

The pH profiles were analysed for gastrointestinal landmarks (ingestion, pylorus, ileocecal valve and excretion) using the following criteria:

▪ Ingestion: rapid and sustained rise in temperature from room to body temperature and a rapid drop of > 3 pH units
▪ Pylorus: rapid and sustained rise of at least 3 pH units
▪ Ileocecal valve (cecum): first and rapid drop of > 0.8 pH units at least 1 h after the pylorus to pH ≤ 6.5
▪ Excretion: rapid and sustained drop in temperature from body to room temperature

Gastrointestinal residence and transit times were derived from the identification of gastrointestinal landmarks and are defined as follows:

▪ Gastric residence time (GRT): elapsed time between ingestion and pylorus
▪ Small intestine transit time (SBTT): elapsed time between pylorus and ileocecal valve
▪ Colonic arrival time (CAT): elapsed time between ingestion and ileocecal valve
▪ Colonic transit time (CTT): elapsed time between ileocecal valve and excretion
▪ Whole gut transit time (WGTT): elapsed time between ingestion and excretion

### Chemicals, isotopes and coated tablets

All substances were of pharmacopoeial grade (Ph. Eur. or USP) and were obtained via a certified wholesaler as described before [6]. The stable isotopes ^13^C-urea and ^15^N_2_-urea (AP 99%) were obtained from an FDA-controlled facility (Isotec, USA). Tablet cores containing 50 mg ^13^C- or 50 mg ^15^N_2_-urea and 25 mg caffeine were compounded in the Department of Hospital and Clinical Pharmacy of the University Medical Center Groningen and analysed according to the European Pharmacopoeia 7^th^ edition

A ColoPulse coating of 13–17 mg/cm^2^ was applied on the tablets containing ^13^C-urea.The coating was composed of a mixture of Eudragit S-100:PEG 6000:Ac-di-sol:talc in a ratio of 7:1:3:2 (w/w). The solvent was an acetone-water 97:3 mixture (w/w). Coating thickness was determined and expressed as the amount of Eudragit S100 applied per cm^2^. Caffeine was added to the ^13^C-urea tablet cores for quality control purposes and was used as a marker substance for the *in vitro* determination of the release characteristics lag- and pulse time in the *in vitro* dissolution test. Caffeine was also added to the ^15^N_2_-urea tablet cores to obtain comparable tablet cores, with no particular function in this tablet. All tablets, coated and uncoated, met established pharmaceutical quality control criteria [6].

### Urea-kinetics

To study the bioavailability from a ColoPulse tablet in the ileo-colonic region the difference in kinetics and fate between ^13^C-urea and ^15^N_2_-urea was used. An overview of the relevant kinetic steps can be found in Maurer et al [23,24]. Release of ^13^C-urea in the ileo-colonic region (urease-rich) from a ColoPulse tablet leads to *in situ* fermentation of ^13^C-urea into ^13^CO_2_ which is subsequently exhaled in breath. The delivery of the isotope in the colon can therefore be established by measuring the ^13^CO_2_ response in breath. Unfermented urea (i.e. release in the small intestine, urease-poor) can be measured as the amount of ^13^C-urea in urine. The second stable isotope of urea, ^15^N_2_-urea, in an uncoated tablet functions as a reference and reflects 100% release in a urease-poor region. Release of ^15^N_2_-urea in the small intestine from an uncoated capsule leads to recovery of ^15^N_2_-urea in urine. Bioavailability can be described by the difference between kinetics of ^13^C- and ^15^N_2_-urea [23,24].

### Sample collections and analysis

Breath samples were collected every 0.5–1 h up to 15 h after intake of the tablets (Table 2) and were analysed as described before [23]. Briefly, ^13^C/^12^C isotope ratios in the CO_2_ of breath samples were analysed by using a validated breath ^13^C-analyser (Thermo Fisher Scientific, Bremen, Germany) based on isotope ratio mass spectrometry (IRMS).

Urine samples were collected during 24 h after intake of the tablets at prescribed intervals (Table 2) in 500 or 1000 mL containers containing an aliquot of 6M HCl. Urine volumes were recorded and 20 mL samples were stored at -80°C until analysis. Concentrations of total ^15^N and ^13^C were determined as described before using an elemental analyzer interfaced with IRMS [23].

### Calculations

The Percentage of the administered Dose Recovered (PDR) of ^13^C and ^15^N in each urine sample, the ratio of the PDRs ^13^C versus ^15^N (the ^13^C/^15^N-ratio), the fermented (F_fermented_) and not-fermented (F_not-fermented_) fraction of ^13^C urea were calculated as described before [23,24]. In short:

▪ F_fermented_ was calculated as the cumulative (c)PDR of ^13^C in breath over a 15 h time period
▪ F_not-fermented_ = cPDR ^13^C / cPDR ^15^N in a 24 h urine collection
▪ Bioavailability = F_fermented_ + F_not-fermented_
▪ The lag time was derived from the cPDR of ^13^C in breath and was defined as de time between administration of the tablets and the time the cPDR reached the value of 5% of cPDR at t = 15 h

All data were corrected for baseline-concentrations of ^13^C and ^15^N in breath and /or urine. Furthermore, breath data were corrected for CO_2_-retention as described before [4].

### Statistical procedures

This study was performed as a bioavailability study. Based on previous data on transit times of a ColoPulse tablet a sample size of 10 patients is needed to detect a clinically relevant difference of 15% between lag-time based on isotope-signal and colon arrival time based on pH with 80% power and a significance level of α = 0.05 (two sided). Because this study was part of another study [6] requiring a higher sample size and anticipating some drop-out 16 subjects were included.

The results were evaluated by descriptive statistics with SPSS version 22. Normal distribution of the data was investigated with the Shapiro-Wilk test. The center was characterized by the mean and standard deviation (pH-data) or the median and corresponding bootstrap based 95% confidence intervals (95% CI). The dispersion was characterized by the coefficient of variation (CV) and range because not all data were normally distributed. A (parametric) paired-samples t-test (two tailed, α = 0.05) was used to compare the results within groups when data were normally distributed for both variables. A (non-parametric) Wilcoxon signed rank test was performed to compare the results when at least one of the variables was not normally distributed. Differences were considered significant when p < 0.05.

### Endpoints

The endpoint was to investigate the relationship between the gastrointestinal pH-profile obtained with the IntelliCap system and release of ^13^C-urea from a ColoPulse tablet and to confirm that release occurs in the ileo-colonic region after pH 7.0 has been reached.

## Results

The results of 15 out of 16 healthy volunteers initially included in the study were evaluated (Fig 1). One volunteer could not swallow the IntelliCap capsule and was therefore excluded without replacement. Two other subjects (6 and 15) appeared to have a prolonged gastric residence time and the IntelliCap capsule was still in the stomach when breakfast and the following meals were taken. Because their gastric residence time was respectively 17 and 22 h data of these subjects were excluded from any analysis comprising transit times. Their lag time based on the isotope signal was within the normal range. Subject 5 was also excluded from analysis comprising lag time and bioavailability, because of a probably altered urea metabolism. This was concluded from the fact that the cPDR ^13^C in breath was <6.5% after 15h combined with a cDPR of unfermented ^13^C in urine of 70% at t = 24 h. The pH profile of this subject was normal with a GRT of 0:15 h and a SBTT of 3.15 h. The coating functioned well, because the appearance of ^13^C in the urine sample could be seen in the sample collected between t = 4 and 7 h and not earlier. This means that ^13^C-urea in stead of being fermented was absorbed into the bloodstream when it was released at the ileo-colonic region. The data from the remaining 12 subjects were available for all analyses.

IntelliCap capsules could be recovered from the feces within 72 hours after intake in 13 out of 15 subjects. For two subjects the temperature data indicated that the IntelliCap capsule had left the body, but the subjects failed to retrieve it from the feces. Absence from the body was confirmed with an abdominal X-ray. No adverse events potentially related to the IntelliCap system were observed during the study.

In three subjects the portable unit ran out of power after circa 60 hours. This did not influence the data collection necessary for the endpoint analysis because in all subjects the IntelliCap capsule already passed the cecum. However, for these subjects time of excretion and whole gut transit time (WGTT) could not be determined.

In three other subjects the communication between the capsule and portable unit was interrupted varying from 4 to 12 h because the subjects did not wear the portable unit close enough to the body or did not wear it. This also did not influence the data collection for endpoint analysis because all interruptions occurred more than 24 h after intake, when the IntelliCap capsule already had passed the cecum. For one of these subjects excretion, CTT and WGTT could not be determined because excretion occurred during the period of interrupted communication. No other loss of data was encountered in the study.

**Fig 2:**
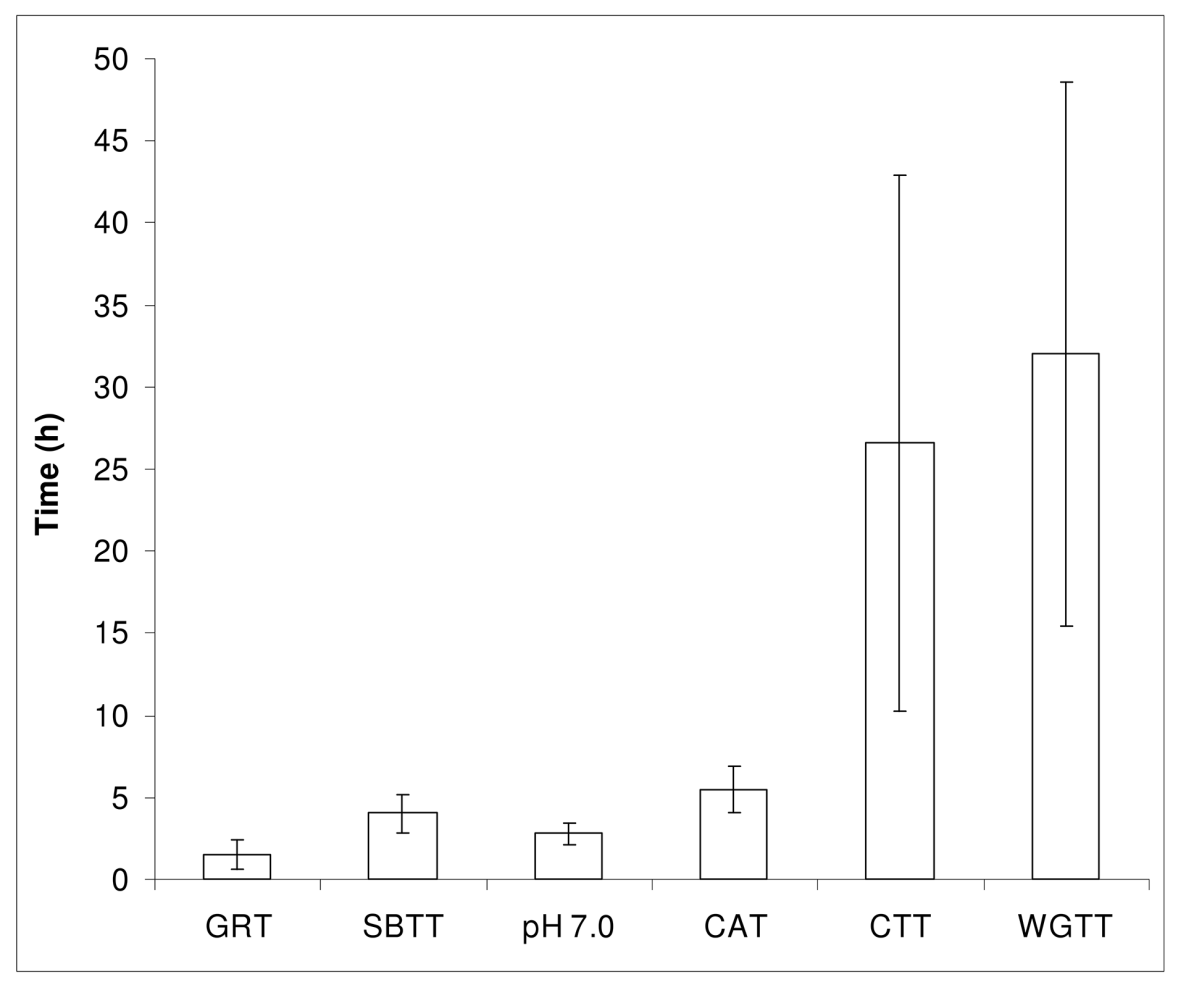
Mean (gastrointestinal residence and transit times determined with the IntelliCap system. Data are presented mean and standard deviation of 13 evaluable subjects (for CTT and WGTT n = 9).

**Fig 3:**
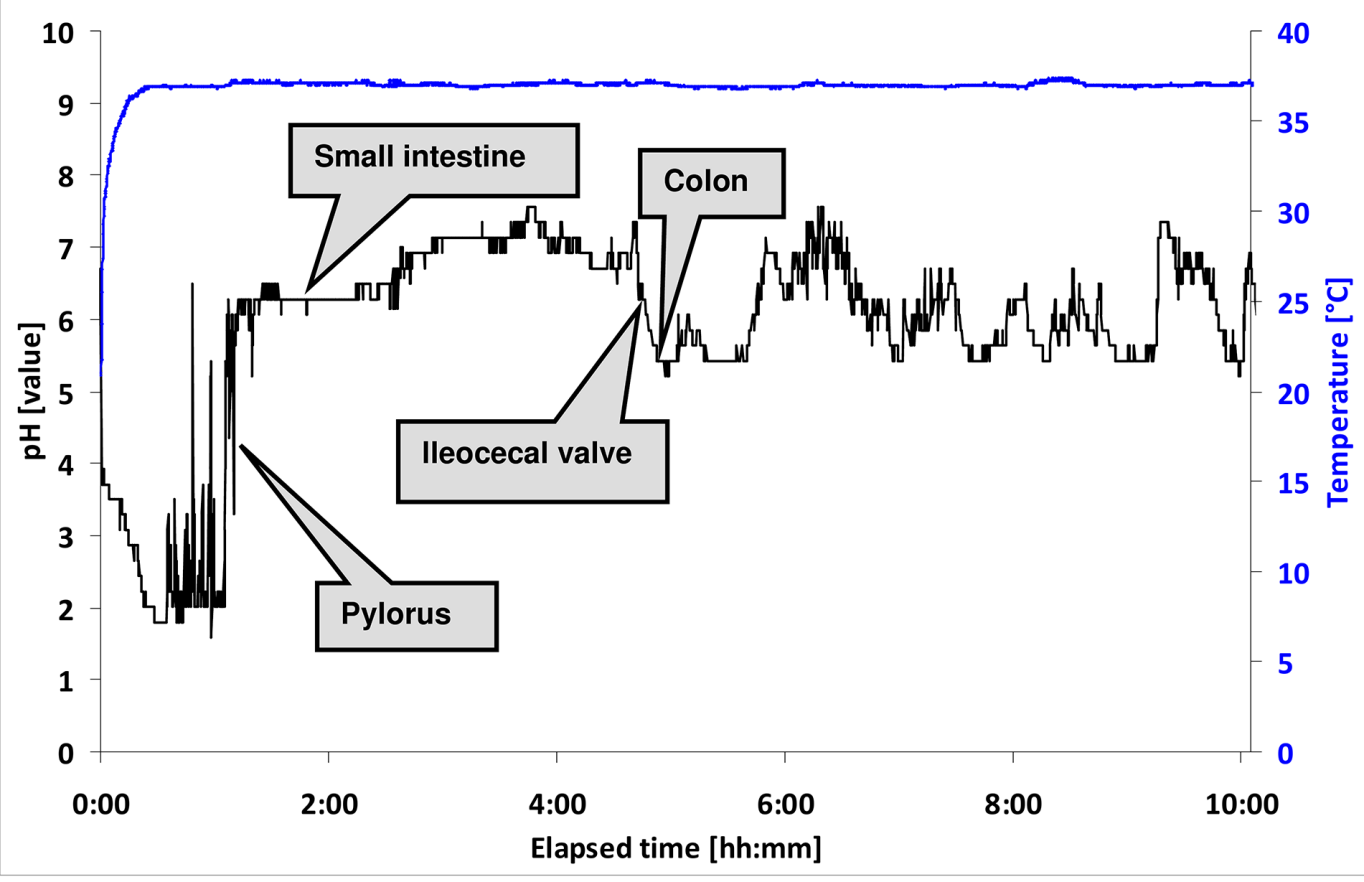
Example of pH-profile of the first 10 hours after intake of the IntelliCap capsule (subject 14).

**Fig 4:**
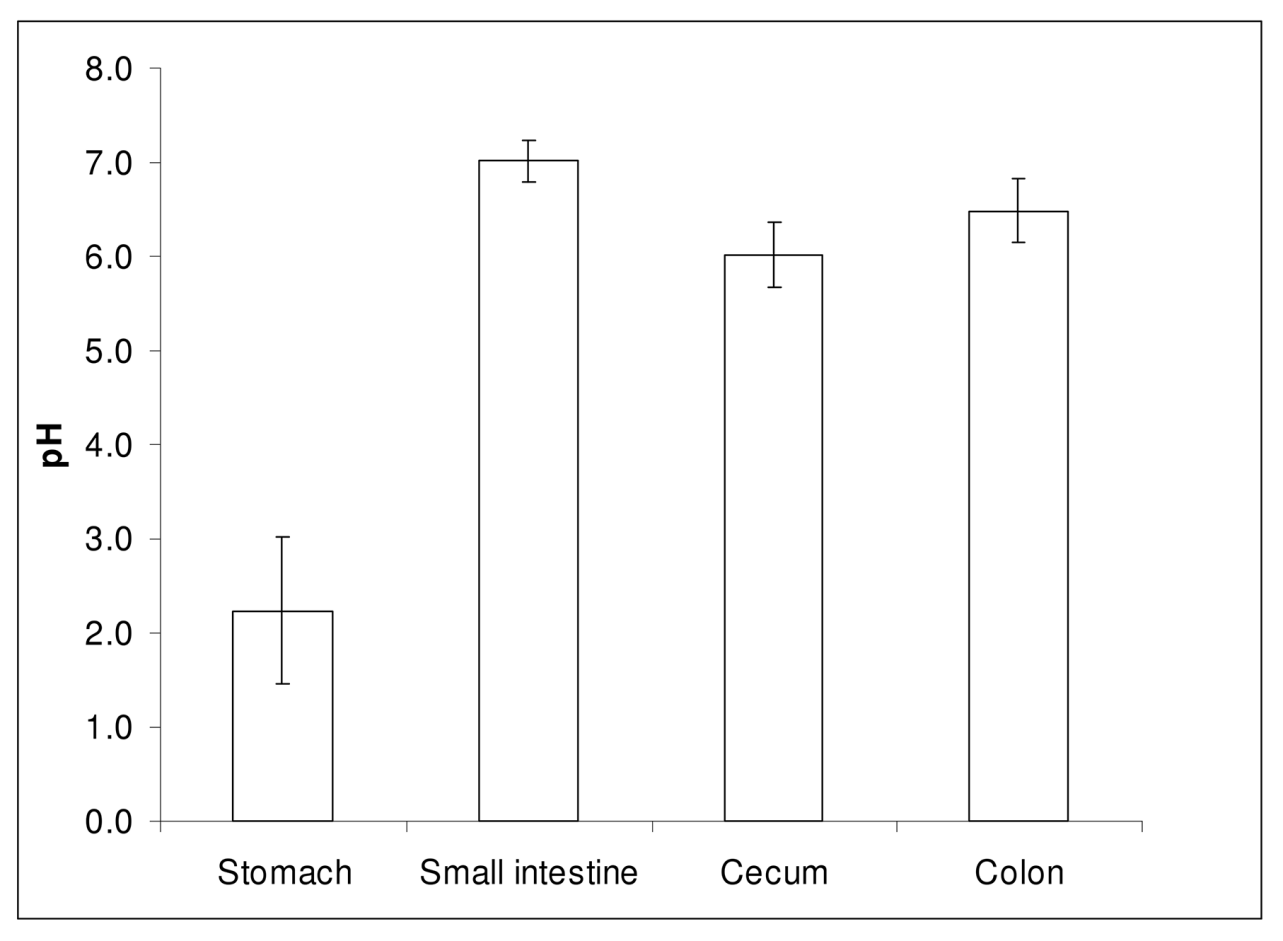
Summary of pH in the stomach, small intestine and colon as measured with the IntelliCap system. Data are presented as mean and standard deviation of 15 evaluable subjects.

All gastrointestinal pH profiles recorded with the IntelliCap system were analysed according to the mentioned methods. A summary of the gastrointestinal transit times is shown in Fig 2 and a representative example of a pH and temperature profile is shown in Fig 3. The residence in the stomach, passage of the pylorus, course of pH in the small intestine and the ileocecal valve (cecum) are all clearly visible in this figure. From Fig 2 it is obvious that there are large inter-individual differences in transit times. For example, the colon arrival time (CAT) differs from 3:25–8:20 h (median 5:31 h, 95% CI 4:51–5:48 h, CV 26%) and the whole gut transit time for the IntelliCap capsule was 10:01–59:39 h (median 27:08 h, 95% CI 22:49–59:11 h, CV 52%). Gastric residence time varied between 0:15 and 3:14 h (median 1:30 h, 95% CI 1:05–2:08 h, CV 59%). The median difference between the time when pH 7.0 was reached and the CAT was 2.26 h and in most subjects pH remained > 7.0 until the cecum was reached. A summary of measured pH values in the stomach, small intestine and colon is shown in Fig 4.

Lag time and bioavailability of ^13^C-urea from a ColoPulse tablet were calculated as described. The median lag time was 5:42 h (95% CI 5:18–6:18 h, range 2:36–6:36 h, CV 18%) and median bioavailability was 82% (95% CI 74–94%) range 61–114%, CV 10%). More detailed results can be found in S1 summary and in Maurer et al [6].

**Fig 5:**
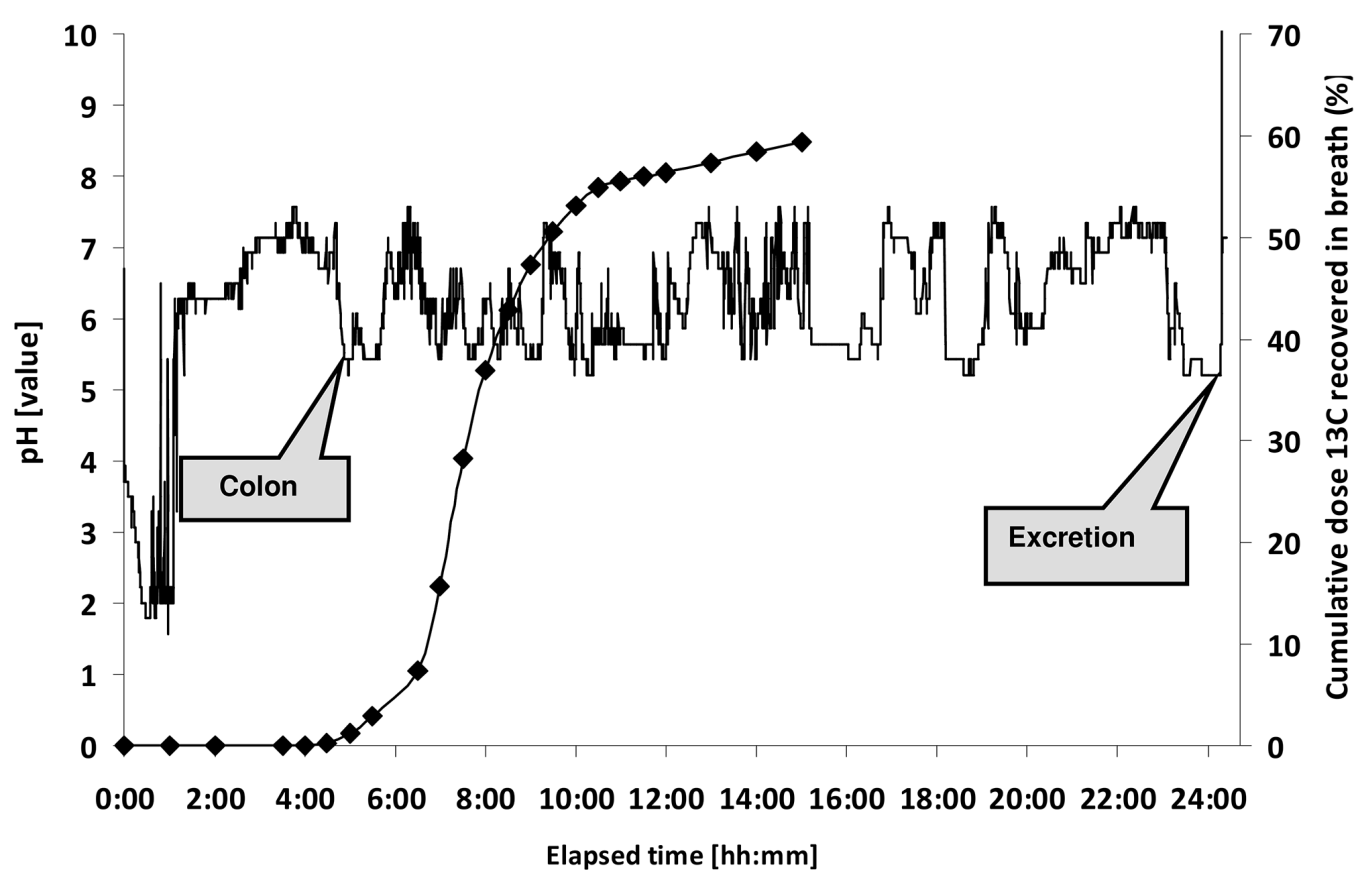
Colon arrival time based on pH (-) corresponds with release of ^13^C (♦) (subject 14). See also Fig 4 for the first 10 hours.

**Fig 6:**
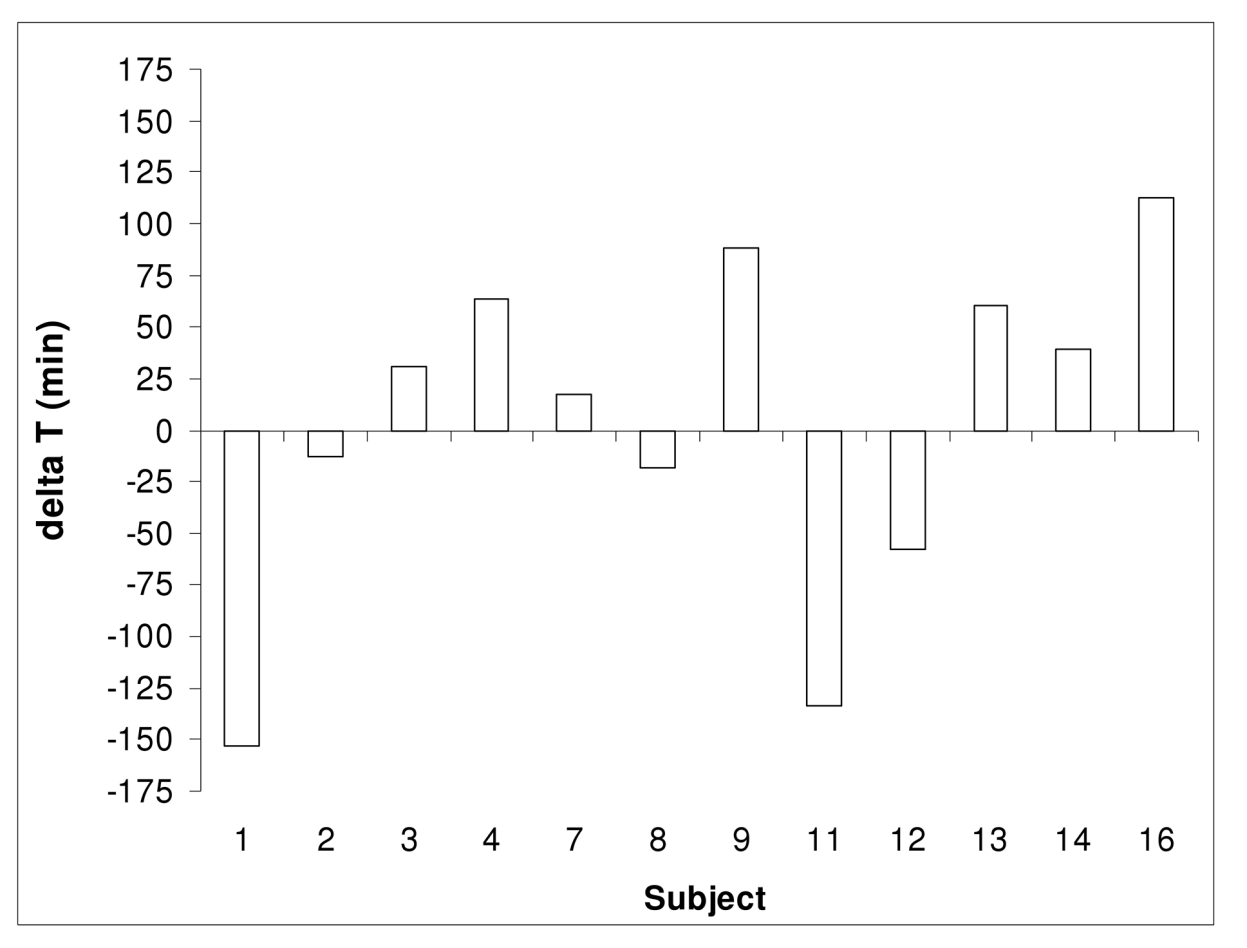
Difference (delta T) between lag-time (based on isotope signal) and CAT (based on pH) for each subject (n = 12).

There was no statistically significant difference between CAT based on pH-data (IntelliCap) and the lag time of the ColoPulse tablet based on the stable isotope signal of ^13^C-urea in breath (median 5:31 vs 5:42 h, p = 0.903, parametric test). A representative example is shown in Fig 5. Information about all subjects can be found in Fig 6.

In all subjects a pH value of 7.0 was reached before release of ^13^C from the ColoPulse tablet occurred, as measured in exhaled breath. There was a statistically significant difference between the time when pH 7.0 was reached and the lag time (185 vs 342 minutes, p = 0.002, non-parametric test).

## Discussion

This is the first study in humans combining release from ColoPulse tablets and *in vivo* gastrointestinal pH measurements using the IntelliCap system. There was no difference between CAT (pH-data) and lag time (isotope data) found in this study which delivers proof that release of ColoPulse tablets occurs in the ileo-colonic region. Furthermore the results show that release from the ColoPulse tablet does not occur before an intestinal pH value of 7.0 is reached.

Several studies have been published with radiotelemetry capsules (RTC). Through the years the functioning of RTCs improved considerably. The first RTCs were used in the 1970’s and had logging intervals from 5 to 120 minutes to save battery capacity [8,10]. Furthermore high data loss (75%) has been described [9]. Thereafter RTCs with shorter logging intervals up to 5 seconds became available and battery life improved considerably [16,17,19]. However in some studies data loss was still described mostly attributed to the angle between the RTC and the antenna [16]. In the current study with the IntelliCap system data loss was observed in three subjects out of 15. This didn’t occur in the first 8 hours after intake of the capsule, when data were also sent to the control center, but only when the subjects were at home and did not keep their receiver close enough to the body. Therefore this event did not influence the outcome of our study, because the IntelliCap capsule already passed the cecum at the time data loss occurred. On the other hand, this shows that an overnight stay in a controlled facility is preferable when longer pH profiling is needed.

The IntelliCap system was able to record complete gastrointestinal pH and temperature profiles as well as derived transit-times from intake to excretion. The observed gastrointestinal transit times for the small intestine and the colon are within the range of earlier published data of healthy volunteers, only the median GRT appeared to be increased [10,25]. Published data collected with RTC and dosage forms labeled with gamma emitting radionuclides mention an average GRT of about ≤ 1 h in fasted, healthy volunteers while we observed an average gastric residence time of 1:30 h in the evaluable subjects. A likely explanation is the intake of the tablets with apple juice, since it is known that food increases the GRT of pharmaceutical dosage forms [25]. Due to its caloric content apple juice apparently also has a delaying effect. However in this study, the apple juice was given to get the same study design as previous studies with ColoPulse formulations. This was done to be able to compare the functioning of ColoPulse tablets used in this study with capsules which were used in previous studies. In the future ColoPulse formulations can also be administered with water.

All subjects showed an elevated gastric pH (pH 3–4) immediately after administration probably due to administration with apple juice, which decreased to a more acidic level of around pH 1.6 in about 30 minutes. Because in our study the determination of the location of the IntelliCap capsule was only based on pH, pH values of the different segments of the small intestine and colon could not be determined. The median pH values of the stomach, small intestines and colon as observed in the majority of subjects are consistent with published data from fasted, healthy volunteers [7].

No difference was found between the colon arrival time based on the ^13^C-isotope signal (lag time of ColoPulse tablets) and pH-measurements (from IntelliCap system). This proves the site-specific release of the active substance from the ColoPulse tablets in the ileo-colonic region. Simultaneous migration of the ColoPulse tablet and the IntelliCap capsule after leaving the stomach is supported by the literature. According to Davis et al [25] no difference in intestinal transit times was seen between solid dosage forms with the same size of ColoPulse tablets and the IntelliCap capsule. Gastric emptying of large single unit systems however, was highly influenced by the presence of food in the stomach. Even a light breakfast delayed emptying in some subjects. This may explain the increased stomach residence time of the IntelliCap capsule in two subjects as seen in the current study. In these subjects colon arrival time and bioavailability based on isotope signal were within the normal range. However the relatively large IntelliCap capsule was retained in the stomach for respectively 17 and 21 h, probably because they returned to their “normal” meal intakes when the IntelliCap capsule was still in the stomach. After pylorus passage of these two IntelliCap capsules intestinal transit times were comparable to those of the other subjects.

In this study we observed no relation between the lag-time of a ColoPulse tablet and the time when pH 7.0 was reached or the CAT. However, in none of the subjects release from the ColoPulse tablet occurred before pH 7.0 was reached.

The difference between the time when pH 7.0 was reached and the CAT was approximately 2.5 hours, supporting the fact that release occurs in the distal ileum and colon. Remarkably, this difference in time is relatively large and differs from parameters used *in vitro* dissolution tests that were performed with the ColoPulse tablets in the gastrointestinal simulation system (GISS) [26]. We use this in vitro test for quality control of ColoPulse tablets and normally dissolution of the coating and subsequent release occurs within 30 minutes after raising pH from 6.8 to 7.5. However, the volumes of intestinal fluid in vivo differ from the volumes used in the GISS. According to Schiller et al [27] the fluid volume of the small intestine has a maximum of 319 mL while the volume in this stage of the GISS is as high as 940 mL. Furthermore the fluid is not distributed homogenously along the small intestine in vivo with water pockets and “dry” segments randomly scattered. This contributes to the relatively slow dissolution of the Eudragit-S coating and is probably the cause of the relatively large difference between the time point at which pH 7 was reached and the CAT. The clinical relevance of this phenomenon seems to be limited because median bioavailability was 82%.

## Conclusion

Based on the combined data from the IntelliCap system and the urea-isotope signal from a ColoPulse tablet as obtained in this study in healthy volunteers it can be concluded that release from ColoPulse tablets indeed occurs in the distal ileum and colon and after pH 7.0 is reached. This supports our earlier observations and confirms that the ColoPulse system is a promising delivery system for site-specific delivery and local therapy in inflammatory bowel diseases present in the distal ileum and colon.

## Supporting Information

S1 Table Summary results of all subjects. (DOC)

S1 File Study protocol (ENG). (DOC)

S2 File Study protocol (NL). (DOC)

S3 File Consort Checklist. (DOC)
