# Supplementary material for: Gastrointestinal pH and Transit Time Profiling in Healthy Volunteers Using the IntelliCap System Confirms Ileo-Colonic Release of ColoPulse Tablets
Source: PLoS One. 2015 Jul 15;10(7):e0129076. doi: 10.1371/journal.pone.0129076 (PMC4503763; doi:10.1371/journal.pone.0129076)
Supplement: S1 Table — (DOC) [file pone.0129076.s001.doc]

S1: summary results of all subjects

|  | IntelliCap system | | | | pH (mean) | | | | Transit times (h:min) | | | | | | Bioavailability (%) | | |
| --- | --- | --- | --- | --- | --- | --- | --- | --- | --- | --- | --- | --- | --- | --- | --- | --- | --- |
| Subject | Swallowing problems | Connection lost | Out of power | IntelliCap  recovered | Stomach | Small  intestine | Cecum | Colon | GRT | SBTT | CAT | CTT | pH 7.0 reached | Lag-time | ^13^C  (breath) | ^13^C/^15^N  (urine) | Total |
| 1 | no | no | yes | yes | 2.03 | 7.45 | 6.28 | 6.51 | 1:33 | 6:18 | 7:51 | n.d. | 3:11 | 5:18 | 75.2 | 38.5 | 113.7 |
| 2 | no | 12h + 9h | no | yes | 1.99 | 7.22 | 6.28 | 6.93 | 1:26 | 4:05 | 5:31 | 54:08 | 3:05 | 5:18 | 14.8 | 69.9 | 84.7 |
| 3 | no | no | yes | yes | 1.77 | 6.98 | 5.58 | 6.28 | 2:10 | 3:13 | 5:23 | n.d. | 3:08 | 5:54 | 53.4 | 41.2 | 94.6 |
| 4 | no | 4h | no | yes | 1.96 | 6.78 | 6.50 | 7.20 | 1:07 | 4:25 | 5:32 | n.d. | 3:19 | 6:36 | 49.5 | 11.4 | 60.9 |
| 5 | no | no | yes | yes | 3.64 | 7.26 | 6.14 | 6.57 | 0.15 | 3:15 | 3:30 | n.d. | 1:26 | n.d. | 6.5 | 70.8 | 77.3 |
| 6 | no | no | no | yes | 1.76 | 7.15 | 5.85 | 6.52 | 21:49 | 4:18 | 26:07 | 9:05 | 23:15 | 7.00 | 60.2 | 16.1 | 76.3 |
| 7 | no | 8.5h + 5.5h | no | yes | 4.15 | 7.09 | 5.92 | 5.97 | 3:14 | 2:59 | 6:13 | 52:58 | 3:14 | 6:30 | 55.9 | 4.9 | 60.8 |
| 8 | no | no | no | yes | 19 | 7.08 | 5.93 | 6.02 | 2:32 | 3:16 | 5:48 | 21:20 | 3:22 | 5:30 | 87.2 | 5.1 | 92.3 |
| 9 | no | no | no | no | 1.78 | 6.86 | 6.07 | 6.51 | 1:30 | 3:02 | 4:32 | 5:29 | 2:53 | 6:00 | 64.6 | 9.7 | 74.3 |
| 10 | yes | n.a. | n.a. | n.a. | n.a. | n.a. | n.a. | n.a. | n.a. | n.a. | n.a. | n.a. | n.a. | 3:06 | 84.8 | 9.2 | 94.0 |
| 11 | no | no | no | no | 1.65 | 7.27 | 6.07 | 6.27 | 2:08 | 6:12 | 8:20 | 22:10 | 3:18 | 6:06 | 63.9 | 13.6 | 77.5 |
| 12 | no | no | no | yes | 1.63 | 6.98 | 6.50 | 6.40 | 1:54 | 3:40 | 5:34 | 17:15 | 2:59 | 4:36 | 65.9 | 19.3 | 85.2 |
| 13 | no | no | no | yes | 1.95 | 6.68 | 6.36 | 6.99 | 0:24 | 4:54 | 5:18 | 20:32 | 2:04 | 6:18 | 53.6 | 17.5 | 71.1 |
| 14 | no | no | no | yes | 2.60 | 6.68 | 5.42 | 6.26 | 1:05 | 3:46 | 4:51 | 19:26 | 2:53 | 5:30 | 59.4 | 31.5 | 90.9 |
| 15 | no | no | no | yes | 1.75 | 6.88 | 5.92 | 6.34 | 17:08 | 5:21 | 22:29 | 12:23 | 19:20 | 5:00 | 90.4 | 5.0 | 95.4 |
| 16 | no | no | no | yes | 3.05 | 6.83 | 5.42 | 6.58 | 0:19 | 3:06 | 3:25 | 25:21 | 1:35 | 5:18 | 60.0 | 19.7 | 79.7 |

n.a. = not applicable

n.d. = could not be determined
