# Supplementary material for: Gastrointestinal pH and Transit Time Profiling in Healthy Volunteers Using the IntelliCap System Confirms Ileo-Colonic Release of ColoPulse Tablets
Source: PLoS One. 2015 Jul 15;10(7):e0129076. doi: 10.1371/journal.pone.0129076 (PMC4503763; doi:10.1371/journal.pone.0129076)
Supplement: S1 File — (DOC) [file pone.0129076.s002.doc]

| **BIOCOP-2: Lokale biobeschikbaarheid ui t een MR tablet (ColoPulse** **®** **-technologie) versie 4, 20101011** **BIOCOP-2: Local bioavailability onion t MR tablet (Colo Pulse** **®** **technology) version 4 20,101,011** |
| --- |

**PROTOCOL BIOCOP - 2 -STUD IE** **PROTOCOL BIOCOP - 2 -STUDY**

**Bio** **beschikbaarheid van een** **Co** **lo** **P** **ulse** **®** **-tablet** **Bioavailability of a** **ColoPulse** **tablet**

(translated version, translation made 09 dec 2014)

**PROTOCOL TITEL :** **PROTOCOL TITLE:**

BIOCOP -2- studie BIOCOP -2- study

**PROTOCOL ONDERTITEL:** **PROTOCOL SUBTITLE:**

Studie naar de lokale biobeschikbaarheid uit een "modified-release" tablet (ColoPulse ® -technologie) bij gezond e vrijwilligers en patiënten . Study on the local bioavailability of a "modified-release" tablet (Colo Pulse ® technology) in healthy volunteers and patients.

| **Korte titel** **Short title** | Colon-specifieke afgifte studie (2) Colon-specific release study (2) |
| --- | --- |
| **Versie** **Version** | 4 .0 4 .0 |
| **Datum** **Date** | 11 -10-2010 11 -10-2010 |
| **EudraCT** **EudraCT** | 2009-013471-21 2009-013471-21 |
| ***METc*** ***METc*** | 2009.188 2009.188 |
| **O nderzoe ks groep** **Research group** | **Drs.** **Drs.** **JM Maurer, ziekenhuisapotheker io** **JM Maurer, hospital pharmacist io**  **Drs.** **Drs.** **HM van Rieke, ziekenhuisapotheker io** **HM Rieke, hospital pharmacist io**  **Drs.** **Drs.** **RCA Schellekens, ziekenhuisapotheker** **RCA Schellekens, hospital pharmacist**  **Dr.** **Dr.** **JGW Kosterink, ziekenhuisapotheker-klinisch-farmacoloog** **JGW Kosterink, hospital pharmacist clinical pharmacologist**  Klinische F armacie & Apotheek Clinical Pharmacie  UMC Groningen UMC Groningen  **Dr.** **Dr.** **G. Dijkstra, gastro-enteroloog** **G. Dijkstra, gastroenterologist**  Interne Geneeskunde Internal Medicine  UMC Groningen UMC Groningen  **Prof.** **Prof.** **Dr.** **Dr.** **HW Frijlink , farmaceutisch technoloog** **HW Frijlink, pharmaceutical technologist**  **Dr.** **Dr.** **HJ Woerdenbag , apotheker** **HJ Woerdenbag, pharmacist**  Farmacie en Farmaceutische Wetenschappen Pharmacy and Pharmaceutical Sciences  Rijksuniversiteit Groningen University of Groningen  **Dr. F.** **Dr. F.** **Stellaard , biochemicus** **Stellaard, biochemist**  Pathologie & Laboratoriumgeneeskunde Pathology and Laboratory Medicine  UMC Groningen UMC Groningen |
| **Sponsor** **Sponsor** | **UMC Groningen** **UMC Groningen**  **Dr.** **Dr.** **JGW Kosterink, ziekenhuisapotheker -klinisch-farmacoloog** **JGW Kosterink, hospital pharmacist –clinical pharmacologist**  Hoofd Klinische F armacie & Apotheek Head of Clinical Pharmacy  UMC Groningen UMC Groningen |
| **Onafhankelijk arts** **Independent physician** | **Drs.** **Drs.** **R . J . Wijdh, oogarts** **R. J. Wijdh, ophthalmologist**  Afdeling Oogheelkunde Department of Ophthalmology  UMC Groningen UMC Groningen |

**PROTOCOL SIGNATURE SHEET** **PROTOCOL SIGNATURE SHEET**

| **Naam** **Name** | **Handtekening** **Signature** | **Datum** **Date** |
| --- | --- | --- |
| Dr. Dr. JGW Kosterink, ziekenhuisapotheker JGW Kosterink, hospital pharmacist  Hoofd Klinische farmacie & Apotheek Head of Clinical Pharmacy  UMC Groningen UMC Groningen |  |  |
| Drs. Drs. RCA Schellekens, ziekenhuisapotheker RCA Schellekens, hospital pharmacist  Klinische farmacie & Apotheek Clinical Pharmacy  UMC Groningen UMC Groningen |  |  |
| Drs. Drs. JM Maurer, ziekenhuisapotheker io JM Maurer, hospital pharmacist io  Klinische farmacie & apotheek Clinical pharmacy  UMC Groningen UMC Groningen |  |  |
| Drs. Drs. HM van Rieke, ziekenhuisapotheker io HM Rieke, hospital pharmacist io  Klinische farmacie & apotheek Clinical pharmacy  UMC Groningen UMC Groningen |  |  |
| Dr. Dr. G. Dijkstra, gastro-enteroloog G. Dijkstra, gastroenterologist  Interne Geneeskunde Internal Medicine  UMC Groningen UMC Groningen |  |  |

Pagina 1 van 21 Page 1 of 21

| **BIOCOP-2: Lokale biobeschikbaarheid ui t een MR tablet (ColoPulse** **®** **-technologie) versie 4, 20101011** **BIOCOP-2: Local bioavailability of a MR tablet (Colo Pulse** **®** **technology) version 4 20,101,011** |
| --- |

**INHOUDSOPGAVE** **TABLE OF CONTENTS**

1. 1.               INTRODUCTIE EN RATIONALE INTRODUCTION AND RATIONALE

2. 2.               STUDIE OPZET STUDY DESIGN

2.1 2.1               Principe Principle

2.2 2.2               Uitvoering studie Study design

3. 3.               STUDIEPOPULATIE STUDY POPULATION

3.1 3.1               Proefpersonen Subjects

3.2 3.2               Inclusiecriteria Inclusion criteria

3.3 3.3               Exclusiecriteria Exclusion

3.4 3.4               Berekening studiegrootte Calculation study size

4. 4.               MEDICATIE MEDICATION

4.1 4.1               Toegepast geneesmiddel Applied medicine

*4.2* *4.2*               Toegepast medisch hulpmiddel Applied medical device

4.3 4.3               Samenvatting informatie uit niet-klinische studies Summary information from non-clinical studies

4.4 4.4               Bijwerkingen Side effects

4.5 4.5               Dosering, toedienvorm en toedienroute Dosage / Dosage form / route of administration

5. 5.               METHODE METHOD

5.1 5.1               Primaire uitkomstmaten Primary outcomes

5.2 5.2               Studieprocedures Study Procedures

5.3 5.3               Tussentijds stoppen Withdrawal

6. 6.               VEILIGHEIDSRAPPORTAGE Safety reporting

6.1 6.1               Sectie 10 WMO Section 10 WMO

6.2 6.2               Risico's proefpersonen Risk subjects

7. 7.               ANALYSE ANALYSIS

7.1 7.1               Analyse Analysis

7.2 7.2               Laboratoriumtechnieken Laboratory Techniques

*7.3* *7.3*               IntelliCap® IntelliCap®

8. 8.               ETHISCHE OVERWEGINGEN ETHICAL CONSIDERATIONS

8.1 8.1               Vereiste verklaringen Required statements

8.2 8.2               Werving en consent Recruitment and consent

8.3 8.3               Verzekering Insurance

8.4 8.4               Vergoeding Compensation

9. 9.               ADMINISTRATIEVE ASPECTEN EN PUBLICATIE ADMINISTRATIVE ASPECTS AND PUBLICATION

9.1 9.1               Archivering studiedocumentatie Archiving study documentation

9.2 9.2               Amendementen Amendments

9.3 9.3               Voortgangsrapportage Progress Report

9.4 9.4               Rapportage studieresultaten Reporting study results

9.5 9.5               Openbaarmaking en publicatie Disclosure and publication

10. 10.               REFERENTIES REFERENCES

**LIJST MET AFKORTINGEN EN RELEVANTE DEFINITIES** **ABBREVIATIONS AND RELEVANT DEFINITIONS**

| **ABR** **ABR** | **Algemene Beoordeling en Registratie formulier** **General Assessment and Registration Form** |
| --- | --- |
| **CV** **CV** | **Curriculum Vitae** **Curriculum Vitae** |
| **IC** **IC**  **IFX** **IFX** | **Informed Conse nt** **Informed Consent**  **Infliximab** **Infliximab** |
| **IMPD** **IMPD** | **Investigational Medicinal Product Dossier** **Investigational Medicinal Product Dossier** |
| **METC** **METC**  **PDR** **PDR**  **TNF** **TNF** | **medisch ethische toetsing commissie (METC)** **medical ethics review committee (METC)**  **Percentage of Dosage Recovered** **Percentage of Dosage Recoverde**  **Tumor Necrose Factor** **Tumor Necrosis Factor** |
| **WMO** **WMO** | **Wet Medisch-wetenschappelijk Onderzoek met Mensen** **Law Medical Research Involving Human Subjects** |

**SamenvattingSSiSi,,**

**Summary Rationale:**

**Rationale:**

Infliximab (IFX) als eerste TNF-alfa remmer heeft de farmacotherapeutische mogelijkheden voor patiënten met de ziekte van Crohn aanmerkelijk verbeterd [1,2].Infliximab (IFX) is a TNF-alpha inhibitor and has therapeutic options for patients with Crohn's disease significantly improved [1,2]. Dit geldt zowel voor de inductie van remissie als het onderhoud daarvan. This applies both to the induction of remission and maintenance thereof. De huidige therapie kent echter een aantal nadelen [1]. The current therapy, however, has a number of disadvantages [1]. Ten eerste, de verbeterde effectiviteit gaat gepaard met het optreden van ernstige bijwerkingen (ernstige infecties zoals tuberculose, hartfalen, auto-immuniteit en maligniteiten). First, the improved efficacy is associated with the occurrence of serious adverse events (serious infections such as tuberculosis, heart failure, autoimmunity and malignancies). Deze bijwerkingen zijn alle gerelateerd aan de systemische blootstelling aan IFX. These effects are all related to the systemic exposure to IFX.

Ten tweede, de conventionele onderhoudsbehandeling (elke 8 weken 5 mg/kg) sluit niet aan op de pathofysiol o gie van de ziekte.Second, the conventional maintenance treatment (every 8 weeks, 5 mg / kg) does not connect it to the pathophysiology of the disease. Dit blijkt onder andere uit de klinische observatie dat een significant aantal patiënten zich reeds eerder dan deze 8 weken meldt voor een nieuw infuus. This is evident from the clinical observation that a significant number of patients already asks earlier than this 8 weeks for a new infusion. Dit fenomeen wordt verklaard uit het feit dat na IFX-behandeling het aantal TNF-alfa producerende cellen in de lamina propria weer langzaam toeneemt , hetgeen leidt tot een toenemende inflammatoire reactie. This phenomenon is explained by the fact that after treatment, the number of TNF-alpha producing cells slowly increases in the lamina propria, resulting in an increased inflammatory response.

Bovenstaande beide nadelen van de onderhoudsbehandeling zijn mogelijk te voorkomen met een lokale en frequentere (dagelijkse) blootstelling van de darmwand aan een lage dosis IFX . The above disadvantages of could possible be prevented with a local and more frequent (daily) exposure of the intestinal wall to a low dose of IFX. In een multi-step project wil de onderzoeksgroep deze benadering verder onderzoeken In a multi-step project, the research group investigates this approach in de betreffende in the respective patiëntpopulatie . In een eerder stadium patient population. At an earlier stage is in de BIOCOP-1 studie is in the 1-BIOCOP study de lokale biobeschikbaarheid van een “ *modified release* ” capsule onderzocht in gezonde vrijwilligers. the local bioavailability of a *"modified release"* capsule investigated in healthy volunteers. Het vervolg van het project bestaat uit de volgende twee delen: The remainder of the project consists of the following two parts:

1. Studie naar de lokale bio beschikbaarheid uit een " *modified-release* " tablet (ColoPulse ® -technologie) bij gezond e vrijwilligers en patiënten met de ziekte van Crohn Study of the local bio availability of a *"modified-release"* tablet (Colo Pulse ® technology), e in healthy volunteers and patients with Crohn's disease **( BIOCOP-2 )** **(BIOCOP-2)**
2. Studie naar de effectiviteit en veiligheid van een " *modified-release* " tablet (ColoPulse ® -technologie) bij patiënten met de ziekte van Crohn **( TOMATE-1 )** . Efficacy and safety of a *"modified-release"* tablet (Colo Pulse ® technology) in patients with Crohn's disease **(TOMATE-1).**

Dit studieprotocol beschrijft het eerste deel , de zogenaamde **BIOCOP - 2** -studie. This study protocol describes the first part, called **BIOCOP - 2** Study.

**Doel:** **Purpose:**

Vaststellen lokale biobeschikbaarheid "modified-release" tablet (ColoPulse ® -technologie) Establish local bioavailability of a"modified-release" tablet (Colo Pulse ® technology) bij gezonde vrijwill i gers en patiënten met de ziekte van Crohn . in healthy volunteers and patients with Crohn's disease.

Vaststellen/opnemen van gastro-intestinale pH profiel bij gezonde vrijwilligers . Determining / recording of gastrointestinal pH profile in healthy volunteers.

**Studie - opzet:** **Study - design:**

Prospectieve biobeschikbaarheidstudie Prospective bioavailability study

**Studiepopulatie** **Study Population**

Proefpersonen: g ezonde vrijwilligers en p atiënten met d e ziekte van Crohn (in remissie) . Test subjects: healthy volunteers and patiënts with Crohn's disease (in remission).

**Interventie** : **Intervention:**

Toedienen ^13^ C-ureum 50 mg modified-release tablet + ^15^ N _2_ - ureum 50 mg immediate release tablet met twee verschillende inname-instructies Administration of ^13^ C-urea 50 mg modified-release tablet + ^15^ N _2_ - urea 50 mg immediate
 release tablet with two different intake instructions en het afnemen van adem en urinemonsters tbv het vaststellen van de biobeschikbaarheid . and the taking of breath and urine
 samples for the determination of the bioavailability.

Inname IntelliCap tbv het vaststelen van het gastro-intestinale pH-profiel . Taking the IntelliCap to establish the gastrointestinal pH profile (healthy volunteers)

**Primaire uitkomstmaten :** **Primary outcomes:**

1. Lokale biobeschikbaarheid , uitgedrukt als de fractie Local bioavailability, expressed as the fraction ^13^ C die in de vorm van ^13^ CO _2_ ^13^ C, which in the form of ^13^ CO _2_ wordt teruggevonden in de adem bij gezonde vrijwilligers en patiënten met de ziekte van Crohn. is recovered in the breath in healthy volunteers and patients with Crohn's disease.
2. Tijd tussen in n ame en respons bij gezonde vrijwilligers en patiënten met de ziekte van Crohn . Dit is het tijdstip waarop de afgifte groter is dan 5% van de maximale cumPDR* van ^13^ C-ureum . . Lagtime in healthy volunteers and patients with Crohn's disease. This is the time when the release is greater than 5% of the maximum cumPDR of ^13^ C-urea..
3. Pulse time bij gezonde vrijwilligers en patiënten met de ziekte van Crohn. Pulse time in healthy volunteers and patients with Crohn's disease. Dit is het verschil tussen de tijdstippen waarop de maximale PDR en de PDR _5%_ wordt bereikt. This is the difference between the lagtime and the time when 70% of the PDR is achieved.
4. Beschrijving van het gastro-intestinale pH-profiel van gezonde vrijwilligers Description of the gastro-intestinal pH-profile of healthy volunteers

**Risico's deelname:** **Risks participation:**

De tabletten bevatten : The tablets contain:

- -           Farmaceutische stoffen, die voor humane toepassing zijn goedgekeurd Pharmaceutical substances, which have been approved for human application

- -           Ureum, een lichaamseigen stof die als marker in kleine Urea, an endogenous substance that as a marker in small hoeveelheden in de vorm van een stabiel, niet radioactie f isoto o p wordt ingenomen . quantities in the form of a
 stable isotope

- -           Coffe ï ne, in een kleine (subtherapeutische) hoeveelheid , die slechts eenmalig wordt ingenomen . Coffeine, in a small (sub-therapeutic) amount that is taken only once.

Retentie van de IntelliCap kan theoretisch gezien optreden, waardoor endoscopische of chirurgische verwijdering noodzakelijk is ( wordt alleen toegepast bij gezonde vrijwilligers).Retention of IntelliCap can theoretically occur, making endoscopic or surgical removal (only applicable to healthy volunteers).

In de “Clinical In vestigator's Brochure” (versie 2 .0) is een literatuuroverzicht gegeven van verschillende, wat betreft afmeting gelijkende, devices, die gebruikt worden bij verschillende toepassingen. In the "Clinical Investigators Brochure" of the IntelliCap (version 2 .0) a review of the literature data of different devices (with similar size), which are used in various applications can be found. Hieruit kan geconcludeerd worden dat er geen capsule retentie is opgetreden bij gezonde vrijwilligers. It can be concluded that no capsule retention occurred in healthy volunteers.

De monstername (adem en urine) geeft geen risico voor de proefpersonen. Sampling (breath and urine) indicates no risk to subjects.

# 1. 1.       INTRODUCTIE EN RATIONALE INTRODUCTION AND RATIONALE

Verbeteren behandeling ziekte van Crohn Improve treatment of Crohn's disease

Infliximab (IFX, Remicade ® ) is een anti-TNF antilichaam dat is geregistreerd voor inductie- en onderhoudsbehandeling van patiënten met therapieresistente ziekte van Crohn (1 maal per 8 weken een dosis van 5 mg/kg intraveneus) . Infliximab (IFX, ® Remicade) is an anti-TNF antibody that has been registered for induction and maintenance treatment of patients with treatment-resistant Crohn's disease (1 times per 8 weeks at a dose of 5 mg / kg intravenously). De effectiviteit van IFX is aangetoond bij zowel de actieve luminale ziekte als bij enterocutane fisteling. IFX kan ook worden toegepast bij extra-intestinale verschijnselen van de ziekte van Crohn zoals pyoderma gangrenosum, uveitis en arthropathie [1] . The effectiveness of IFX has been demonstrated in both the active luminal disease as in enterocutaneous fistula. IFX can also be used in extra-intestinal manifestations of the disease including pyoderma gangrenosum, Crohn's disease and uveitis [1]. Recent is aangetoond dat vroege behandeling met IFX mogelijk effectiever is dan de standaard step-up behandeling waarbij alleen therapieresistente patiënten dit middel krijgen toegediend [2]. Afgezien van het wegvangen van anti-TNF wordt de effectiviteit van IFX en adalimumab vooral toegeschreven aan het vermogen om geactiveerde mucosale T- cellen waarbij TNF op het celoppervlak aanwezig is Recently it has been shown that early treatment with IFX is more effective than the standard step-up treatment in which only treatment-resistant patients get this agent is administered. [2] Apart from scavenging anti-TNF is the effectiveness of IFX and adalimumab mainly attributed to the ability to activated mucosal T-cells in which TNF is present on the cell surface tot apoptose te brengen. to bring to apoptosis. Na een infuus komen deze cellen echter terug waardoor een onderhoudsbehandeling en combinatietherapie met afweerremmende middelen (azathioprine, methotrexaat) noodzakelijk wordt. After infusion, these cells are back creating a maintenance treatment and combination therapy with immunosuppressive agents (azathioprine, methotrexate) is necessary. Een probleem van de intermitterende intraveneuze of subcutane toediening vormen de infuusreacties en loss of response. A problem of the intermittent intravenous or subcutaneous administration are the infusion reactions and loss of response. Loss of response is veelal geassocieerd met de vorming van anti- IFX antistoffen. Daarnaast is er vanwege de systemische expositie van IFX meer kans op systemische bijwerkingen waarbij infecties en in het bijzonder tuberculose het meest gevreesd worden. Om systemische expositie te vermijden , zijn er met name bij fistels studies gedaan met locale injectie van IFX . Loss of response is often associated with the formation of anti-IFX antibodies. In addition, there is because of the systemic exposure of IFX greater risk of systemic side effects involving infections and, in particular tuberculosis are most feared. In order to avoid systemic exposure, there are with especially in fistula studies with local injection of IFX. Bij de ziekte van Crohn is de darmmucosa vooral In Crohn's disease, the intestinal mucosa is especially ter plaatse van de ontsteking verhoogd permeabel. at the location of the inflammation enhanced permeable. De ontsteking zit meestal in het terminale ileum en colon. Mogelijk zou f requentere en/of continue lo k ale delivery van een anti-TNF middel ter plekke van de ontsteking de voorkeur genieten boven de 8-weekse The inflammation is usually located in the terminal ileum and colon. Possibly a more frequent or continuous local delivery of an anti-TNF agent at the site of inflammation will be more preferable compared to the 8-week intraveneuze toediening vanwege de lagere systemische belasting . Daarnaast zou intravenous administration because of the lower systemic loading. In addition, would dagelijkse lokale delivery van een lage re keer dos is daily local delivery of a low dosis continuous apoptosis induction may cause developing T cells, which better reflects the pathophysiological processes. Verder zijn er minder systemische bijwerkingen en anti-stofvorming te verwachten en hoeft patiënt geen infusen of injecties meer de ondergaan. Kortom, de benefit-risk van IFX-behandeling zou verbeteren, hetgeen bij de ziekte van Crohn een doorbraak beteken t . Furthermore, there are likely fewer systemic side effects and patient do not have to be exposed to infusions or injections anymore. In short, it would improve the benefit-risk of IFX treatment, which means a breakthrough in Crohn's disease.

Ontwikkeling toedieningsvorm Development dosage form

In een samenwerkingsverband van UMCG en R U G is een technologie ontwikkeld waarmee na orale inname van In a collaboration of UMCG and R U G a technology has been developed that allows after oral intake ofeen tablet of capsule selectieve afgifte van geneesmiddelen kan plaatsvinden in het terminale ileum / proximale colon [3,4,5] . a tablet or capsule selective delivery of drugs in the terminal ileum / proximal colon [3,4,5]. Voordat een patiëntenstudie Before a patient study naar de uitkomsten van to the results of topicale behandeling met oraal IFX k an plaatsvinden, moet eerst gevalideerd worden dat het afgifteprofiel van de te gebruiken formulering juist is. De opzet van deze studie bouwt op de resultaten en ervaring opgedaan in studie CO-06-01 (BIOCOP-1) : “ Pilotstudie naar afgifte uit een orale, colon specifieke, afgifte formulering in gezonde vrijwilligers met behulp van stabiele isotopen ” . topical treatment with oral IFX can take place, it must be validated the release profile of the formulation used is correct. The purpose of this study builds on the results and experience gained in the study of CO-06-01 (BIOCOP-1). " Pilot study to release from an oral, colon specific release formulation in healthy volunteers using stable isotopes ". In deze studie werd de geschiktheid van isotopisch ureum als markerstof bestudeerd [6]. In this study, the suitability of isotopically urea was studied as a marker substance [6]. Ook werd in deze studie de lokale biobeschikbaarheid van een gecoate capsule onderzocht in gezonde vrijwilligers [7]. Also the local bioavailability of a coated capsule was studied in healthy volunteers [7]. Ten behoeve van de IFX-interventie is een tablet ontwikkeld, waarvan het afgifteprofiel gevalideerd moet zijn , zowel in gezonde vrijwilligers als in patiënten met de ziekte van Crohn [8] . D it is het doel van de For the purpose of the IFX-intervention a tablet has been developed of which the release profile to be validated, in both healthy subjects and in patients with Crohn's disease. [8] This is the purpose of the **BIOCOP-2** -studie . **BIOCOP-2** bioavailability study.

Om aanvullende informatie te verkrijgen over de werking van de pH-gevoelige coating van het "modified-release" tablet, wordt eenmalig het gastro-intestinale pH-profiel van gezonde vrijwilligers bepaald met behulp van de IntelliCap®. In order to obtain additional information about the functioning of the pH-sensitive coating of the "modified-release" tablet, the gastro-intestinal pH-profile of healthy volunteers will be determined with the help of the determined IntelliCap®.

De IntelliCap® wordt geproduceerd door Philips (zie bijlage 1).

The IntelliCap® is produced by Philips Medimetrics (see Annex 1). Dit is een medical device in de vorm van een capsule (afmeting: 18mm bij 11mm), dat oraal wordt ingenomen, de pH in het maagdarmkanaal meet, en wordt uitgescheiden met de ontlasting. *De IntelliCap® wordt gevuld met fysiologisch zout (NaCl 0,9%).* This is a medical device in the form of a capsule (size: 18mm by 11mm), which is taken orally, measuring the pH in the gastrointestinal tract and is excreted with the feces. the IntelliCap® is filled with physiological saline (NaCl 0,. 9%). Het is van belang de pH in het maagdarmkanaal te volgen, omdat de desintegratie van de “modified-release” tablet afhankelijk is van de pH. It is important to monitor the pH in the gastrointestinal tract, because the disintegration of the "modified-release" tablet is dependent on the pH. Inzicht in de pH kan eventuele non-respons op de ontwikkelde tabletten verklaren. Understanding the pH can explain any non-response to the developed tablets. Het meten van de pH in het maagdarmkanaal met behulp van een capsule is reeds eerder in de literatuur beschreven [9-11]. The measurement of the pH in the gastrointestinal tract with the aid of a capsule has already been described in the literature [9-11].

**DOELSTELLINGEN** **OBJECTIVES**

Vaststellen dat het afgifteprofiel van de tabletformulering voldoet bij gezonde vrijwilligers ( **BIOCOP-2 A** ) en patiënten ( **BIOCOP-2B** ). To determine that the release profile of the tablet formulation does not differ in healthy
 volunteers **(BIOCOP-2 A)** and patients **(BIOCOP-2B).**

Vaststellen van het gastro-intestinale pH-profiel bij gezonde vrijwilligers ( **BIOCOP-2A** ) . Determining the gastrointestinal pH profile in healthy volunteers **(BIOCOP-2A).**

# 2. 2.       STUDIE OPZET STUDY DESIGN

## 2.1 2.1          **Principe** **Principle**

De lokale biobeschikbaarheid van het "modified-release" tablet (ColoPulse ® -technologie) [5] wordt onderzocht met behulp van ureum als marker [ 12 ] . The local bioavailability of the "modified-release" tablet (Colo Pulse ® technology) [5] is examined with the aid of urea as a marker [12]. In essentie wordt dezelfde benadering toegepast als in de **BIOCOP-1** -studie. Essentially the same approach is applied as in the **BIOCOP-1** External Form of Study.

Een "immediate release" tablet 50 mg ^15^ N _2_ -ureum en een "modified-release" tablet 50 mg ^13^ C-ureum worden tegelijk oraal ingenomen . De tablet 50 mg ^15^ N _2_ -ureum zal reeds in de An "immediate release" tablet 50 mg ^15^ N _2_ urea and a "modified-release" tablet 50 mg ^13^ C-urea be taken together orally. The tablet 50 mg ^15^ N _2_ urea will already disintegrate in the maag desintegreren en de ^15^ N _2_ -ureum wordt snel en volledig opgenomen. stomach and ^15^ N _2_ urea is rapidly and completely absorbed. Uitscheiding geschiedt voor het overgrote deel renaal via de urine. Excretion will be in the urine. De biobeschikbaarheid van ^15^ N _2_ -ureum zal worden gebruikt als referentie om de hoeveelheid opgenomen ^13^ C uit ^13^ C-ureum te bepalen. The bioavailability of -urea ^15^ N _2_ will be used as the reference to determine the amount of i^13^ C from ^13^ C-urea.

De tablet 50 mg ^13^ C-ureum zal niet desintegreren in de maag en de dunne darm vanwege de aangebrachte coating (ColoPulse ® -technologie). The tablet 50 mg of ^13^ C-urea will not disintegrate in the stomach and the small intestine due to the applied coating (Colo Pulse ® technology). In het terminale ileum d e sintegreert de tablet op basis van de lokale zuurgraad in dat darmsegment. The tablet will disintegrate in the terminal ileum / colon based on the local pH in the intestine segment. De ^13^ C-ureum komt in het terminale ileum en colon beschikbaar en wordt door het bacteriële urease gehydrolyseerd tot ^13^ CO _2_ en NH _3_ . The ^13^ C-urea will be released in the terminal ileum and colon and will be hydrolyzed to ^13^ CO _2_ and NH _3_ bacterial ureases.

H _2_ N- ^13^ C-NH _2_ + H _2_ O → 2 NH _3_ + ^13^ CO _2_ H _2_ N- ^13^ C-NH _2_ + H _2_ O → 2 NH _3_ + ^13^ CO _2_

Het vrijgekomen ^13^ CO _2_ wordt geabsorbeerd in de circulatie en wordt als ^13^ C-bicarbona at naar de longen vervoerd, waar het dan in de vorm van ^13^ CO _2_ vr ijkomt in de uitgeademde lucht.The released ^13^ CO _2_ is absorbed into the circulation, and is as as ^13-^C -bicarbonate transported to the lungs, where it is exhaled as ^13^ CO _2_.

Bij opening van de tablet in een eerder stadium (dunne darm) wordt ^13^ C-ureum snel en nagenoeg volledig opgenomen in de circulatie .Upon opening of the tablet at an earlier stage (small intestine) is ^13^ C-urea is rapidly and almost completely absorbed into the circulation. Het wordt dan op een zelfde wijze als ^15^ N2-ureum verdeeld en renaal geëlimineerd en uitgescheiden via de urine. It is then excreted in the same way as ^15^ N2 urea and renal eliminated and excreted through the urine. Door in adem ^13^ CO _2_ en in urine ^15^ N en ^13^ C te kwantificeren kan de lokale biobeschikbaarheid van het "modified-release" tablet (ColoPulse ® -technologie) berekend worden naar analogie van berekeningen zoals die gebruikt werden in de **BIOCOP-1** -studie. By analysis of ^13^ CO _2_ in breath and in urine to quantify ^15^ N and ^13^ C, the local bioavailability of the "modified-release" tablet (Colo Pulse ® technology) can be calculated by analogy of calculations, such as those used in the **BIOCOP-1** - study.

Van de gezonde vrijwilligers worden een pH-profiel opgenomen met behulp van de IntelliCap®.In In In healthy volunteers, a pH profile will be recorded with the IntelliCap®. Daarvoor draagt de gezonde vrijwilliger een data-recorder (kastje) om z'n middel, dat de pH-waarden registreert die de IntelliCap® uitzendt. Therefore the healthy volunteer carries a data recorder around his waist, which records the pH values ​​that the IntelliCap transmits®. De IntelliCap® meet de pH iedere 10 seconden. The IntelliCap® measures the pH every 10 seconds. Het kastje met gegenereerde pH-waarden wisselt de data ook iedere 10 seconden uit met het software programma op de computer.

## 2.2 2.2          **Uitvoering studie** **study design**

De studieopzet voor **BIOCOP-2A** en **BIOCOP-2B** is gelijk. The study design for **BIOCOP-2A** and **BIOCOP-2B** is almost equal. De proefpersonen zullen The subjects will administer the same tablets on both test days with washout period of at least 7 days. These test days will be referred at as test day 1 and test day 2 in this Protocol*.* Op testdag 1 wordt na 1 uur een zelf-gekozen ontbijt genuttigd. On test day 1 a self-selected breakfast is consumed after 1 hour. Op testdag 2 wordt na 3 uur een gestandaardiseerd ontbijt genuttigd . Hiermee kan een beeld worden gekregen van de mate waarin tijdstip en vorm van ontbijt een rol spelen bij de biologische beschikbaarheid uit de modified-release tablet . Deze gegevens zijn noodzakelijk voor vervolgonderzoek . Op testdag 2 nemen de gezonde vrijwilligers (BIOCOP-2A) tevens de IntelliCap® in. On test day 2 a standardized breakfast is consumed after 3 hours. This will give information to the extent of which time and type of breakfast play a role in the bioavailability of the modified-release tablet. These data are necessary for further research. On test day 2, the healthy volunteers (BIOCOP-2A) also will swallow the IntelliCap®.

Op beide testdagen worden twee On both test days, two tabletten tegelijk ingenomen op de testlocatie : tablets taken at once at the test site:

- -           1 "modified-release" 1 "modified-release" tablet tablet 50 mg ^13^ C-ureum 50 mg ^13^ C-urea

- -           1 "immediate release" tablet 1 "immediate release" tablet 5 0 mg ^1 5^ N _2_ -ureum 50 mg of ^15^N _2_ urea

De volgende toedieninstructie zal worden gegeven: neem beide tabletten in om 8.00 uur 'sThe following instructions will be given: take two tablets and the intellicap (when applicable) at 8.00 am with 150 mL appelsap . Op test dag 1 wordt na 1 uur eenapple juice. On test day 1, after 1 hour, a zelf-gekozen ontbijt genuttig d . Op test dag 2 wordt na 3 uur een gestandaardiseerd ontbijt genuttigd . Naast de in tabel 1 en 2 omschreven hoeveelheden vocht self-selected breakfast can be chosen. On the test day 2 a standardized breakfast will be served after 3 hours. In addition to the quantities specified in Table 1 and 2, drinks (water, appelsap of thee zonder suiker en melk) en tijdstippen van de maaltijd (Water, juice or tea without sugar and milk) and times of the meal is nuttigen is consuming van voeding niet aan instructies gebonden. food not bound by any limitations. Participants can take to their preferences additional moisture in the form of water, juice or tea without sugar and milk. After taking the tablets and urine and breath samples will be taken according to a fixed schedule. (see Table 1 and 2).

Summary

- - De proefpersonen mogen vanaf 20.00 uur 's avonds tot de ochtend voor de test geen vast voedsel meer innemen.The subjects are not allowed to take solid food from 20.00 pm until the morning of the test day Drinking water, juice or tea without sugar and milk is allowed.
- -Op de testdag nemen de proefpersonen de tabletten in op de testlocatie en wordt gestart met de monstername van adem en urine.On the test day, the subjects take the tablets at the test site and where sampling of breath and urine starts
- -Op de tweede testdag nemen de gezonde vrijwilligers de IntelliCAP®, gelijktijdig met de tabletten, in op de testlocatie.On the second test day the healthy volunteers take IntelliCap® simultaneously with the tablet, at the test site. Daarbij dragen zij om hun middel een kastje (data-recorder) dat de pH-waarden opvangt. In addition they wear around their waist a data recorder that record the pH values until excretion of the IntelliCap. De gezonde vrijwilligers nemen het kastje mee naar huis; Healthy volunteers take the data recorder at home
- -Around 17:00, the subject can go home and can take a chosen dinner. De hele avond worden nog adem- en urinemonsters verzameld tot het slapen en wordt geen vast voedsel ingenomen. The whole evening breath and urine samples are collected by the subject according to the schedule. Drinking water, juice or tea without sugar and milk is allowed.
- -The day after the test at 8.00 am still gathered one last breath and urine sample. After this the subject can have breakfast
- -The IntelliCap® should be collected from the stool and should be handed over to the researcher in a collection container.

the

**Tabel 1: schema voor monstername en maaltijd inname test dag 1 (inname 1 uur voor ontbijt)** **Table 1: schedule for sampling and meal intake test day 1 (intake 1 hour before breakfast)**

| **Dag** **Day** | **Tijd** **Time** | **Actie** **Action** | **Test 1: Ademmonster** **Test 1: Breath Sample**  **( Testlocatie )** **(Test site)** | **Test 1:** **Test 1:**  **Ademmonster** **Breath Sample**  **(Thuis)** **(Home)** | **Test 1:** **Test 1:**  **Urinemonster** **Urine Sample**  **(Thuis)** **(Home)** | **Test 1:** **Test 1:**  **Urinemonster** **Urine Sample**  **( Testlocatie** ***)*** **(Test site*)*** | **Vocht** **Moisture** |
| --- | --- | --- | --- | --- | --- | --- | --- |
| 0 0 | 20. 00 20. 00 | Vasten Fast |  |  |  |  |  |
| 1 1 | 8. 00 8. 00 | Monstername Sampling | X X |  |  | X X  (na opstaan) (After getting up) |  |
|  | 8. 00 8. 00 | Tabletten innemen Take tablets |  |  |  | X X | 150 mL 150 mL |
|  | 9. 00 9. 00 | Monstername Sampling | X X |  |  |  |  |
|  | 9. 00 9. 00 | Ontbijt Breakfast |  |  |  |  | 150 ml 150 ml |
|  | 9. 30 9. 30 | Monstername Sampling | X X |  |  |  |  |
|  | 10. 00 10. 00 | Monstername Sampling | X X |  |  |  |  |
|  | 10 . 30 10. 30 | Monstername Sampling | X X |  |  |  | 150 ml 150 ml |
|  | 11 . 00 11. 00 | Monstername Sampling | X X |  |  |  |  |
|  | 1 1. 30 1 1. 30 | Monstername Sampling | X X |  |  |  |  |
|  | 1 2. 00 1 2. 00 | Monstername Sampling | X X |  |  |  |  |
|  | 1 2. 30 1 2. 30 | Monstername Sampling | X X |  |  | X X |  |
|  | 13.00 13:00 | Monstername Sampling | X X |  |  |  |  |
|  | 13.00 13:00 | Lunch Lunch |  |  |  |  | 150 ml 150 ml |
|  | 13:30 13:30 | Monstername Sampling | X X |  |  |  |  |
|  | 14.00 14:00 | Monstername Sampling | X X |  |  |  | 150 ml 150 ml |
|  | 14.30 14:30 | Monstername Sampling | X X |  |  |  |  |
|  | 15.00 15:00 | Monstername Sampling | X X |  |  | X X |  |
|  | 15.30 15:30 | Monstername Sampling | X X |  |  |  | 150 ml 150 ml |
|  | 16.00 16:00 | Monstername Sampling | X X |  |  |  |  |
|  | 16.30 16:30 | Monstername Sampling | X X |  |  |  | 150 ml 150 ml |
|  | 17.00 17:00 | Monstername Sampling | X X |  |  |  |  |
|  | 17.30 17:30 | Monstername Sampling |  | X X | X X |  |  |
|  | 18.00 18:00 | Monstername Sampling |  | X X |  |  |  |
|  | 18.30 18:30 | Diner (rond dit tijdstip) Dinner (around this time) |  |  |  |  |  |
|  | 18.30 18:30 | Monstername Sampling |  | X X |  |  |  |
|  | 19.00 19:00 | Monstername Sampling |  | X X |  |  |  |
|  | 19.30 19:30 | Monstername Sampling |  | X X |  |  |  |
|  | 20.00 20:00 | Monstername Sampling |  | X X | X X |  |  |
|  | 21.00 21:00 | Monstername Sampling |  | X X |  |  |  |
|  | 22.00 22:00 | Monstername Sampling |  | X X |  |  |  |
|  | 23.00 23:00 | Monstername Sampling |  | X X |  |  |  |
| *2* *2* | 8.00 8:00 | Monstername Sampling |  | X X | X X |  |  |
|  | 8.05 8:05 | Ontbijt Breakfast |  |  |  |  |  |

**Tabel 2: schema voor monstername en maaltijd inname test dag 2 (inname 3 uur voor ontbijt)** **Table 2: schedule for sampling and meal intake test day 2 (taking 3 hours for breakfast)**

| **Dag** **Day** | **Tijd** **Time** | **Actie** **Action** | **Test 2: Ademmonster** **Test 2: Breath Sample**  **(Testlocatie)** **(Test site)** | **Test 2:** **Test 2:**  ***Ademmonster*** ***Breath Sample***  **(Thuis)** **(Home)** | **Test 2:** **Test 2:**  **Urinemonster** **Urine Sample**  **(Thuis)** **(Home)** | **Test 2:** **Test 2:**  **Urinemonster** **Urine Sample**  **(Testlocatie)** **(Test site)** | **Vocht** **Moisture** |
| --- | --- | --- | --- | --- | --- | --- | --- |
| 0 0 | 20. 00 20. 00 | Vasten Fast |  |  |  |  |  |
| 1 1 | 8. 00 8. 00 | Monstername Sampling | X X |  |  | X X |  |
|  | 8. 00 8. 00 | Tabletten Tablets en indien van toepassing IntelliCap® and, where applicable IntelliCap® innemen occupy |  |  |  | X X | 150 mL 150 mL |
|  | 9. 00 9. 00 | Monstername Sampling | X X |  |  |  |  |
|  | 10. 00 10. 00 | Monstername Sampling | X X |  |  |  |  |
|  | 11. 00 11. 00 | Ontbijt Breakfast |  |  |  |  | 150 ml 150 ml |
|  | 11. 30 11. 30 | Monstername Sampling | X X |  |  |  |  |
|  | 12. 00 12. 00 | Monstername Sampling | X X |  |  |  |  |
|  | 12.30 12:30 | Monstername Sampling | X X |  |  | X X | 150 ml 150 ml |
|  | 13.00 13:00 | Monstername Sampling | X X |  |  |  |  |
|  | 13.30 13:30 | Monstername Sampling | X X |  |  |  |  |
|  | 14.00 14:00 | Lunch Lunch |  |  |  |  | 150 ml 150 ml |
|  | 14.30 14:30 | Monstername Sampling | X X |  |  |  |  |
|  | 15.00 15:00 | Monstername Sampling | X X |  |  | X X |  |
|  | 15.30 15:30 | Monstername Sampling | X X |  |  |  | 150 ml 150 ml |
|  | 16.00 16:00 | Monstername Sampling | X X |  |  |  |  |
|  | 16.30 16:30 | Monstername Sampling | X X |  |  |  | 150 ml 150 ml |
|  | 17.00 17:00 | Monstername Sampling | X X |  |  |  |  |
|  | 17.30 17:30 | Monstername Sampling |  | X X | X X |  |  |
|  | 18.00 18:00 | Monstername Sampling |  | X X |  |  |  |
|  | 18.30 18:30 | Diner (rond dit tijdstip) Dinner (around this time) |  |  |  |  |  |
|  | 18.30 18:30 | Monstername Sampling |  | X X |  |  |  |
|  | 19.00 19:00 | Monstername Sampling |  | X X |  |  |  |
|  | 19.30 19:30 | Monstername Sampling |  | X X |  |  |  |
|  | 20.00 20:00 | Monstername Sampling |  | X X | X X |  |  |
|  | 21.00 21:00 | Monstername Sampling |  | X X |  |  |  |
|  | 22.00 22:00 | Monstername Sampling |  | X X |  |  |  |
|  | 23.00 23:00 | Monstername Sampling |  | X X |  |  |  |
| *2* *2* | 8.00 8:00 | Monstername Sampling |  | X X | X X |  |  |
|  | 8.05 8:05 | Ontbijt Breakfast |  |  |  |  |  |

# 3. 3.       STUDIEPOPULATIE STUDY POPULATION

## 3.1 3.1          Proefpersonen Subjects

De studie wordt uitgevoerd met 16 gezonde vrijwilligers in **BIOCOP-2A** . The study is conducted with 16 healthy volunteers **BIOCOP-2A.**

De studie wordt uitgevoerd met 16 patiënten in **BIOCOP-2B** . The study is conducted with 16 patients in **BIOCOP-2B.**

## 3.2 3.2          Inclusiecriteria Inclusion criteria

              Gezonde vrijwilligers Healthy volunteers

- Wilsbekwame, gezonde volwassenen (18-65 jaar) Healthy adults (18-65 years) able to sign informed consent
- Geen medicatiegebruik gedurende de laatste 3 maanden van geneesmiddelen No medication use during the last 3 monthsdie de gastrointestinale flora k unnen that can influence the gastrointestinal
   flora (e.g. antibiotics)
- Geen medicatiegebruik gedurende de laatste 4 weken van geneesmiddelen die de gastrointestinale passagetijd kan beïnvloeden (bijv. laxantia, maagzuurremmers , darmtonus beïnvloedende No medication use during the last 4 weeks of drugs that may affect the
   gastrointestinal transit time (eg. Laxatives, antacids
- Geen gebruik van NSAID's gedurende de laatste 4 weken No use of NSAIDs during the last 4 weeks

              Patiënten Patients

- Wilsbekwame, volwassenen met de ziekte van Crohn (in remissie Adults (18-65 years) able to sign informed consent with Crohn's disease (in
   remission Harvey Bradshaw Index ≤3) (18-65 jaar) Harvey Bradshaw index ≤3) (18-65)
- Geen medicatiegebruik gedurende de laatste 3 maanden van geneesmiddelen die de gastrointestinale flora kunnen beïnvloeden (bijv antibiotica) No medication use during the last 3 months of drugs that may affect the
   gastrointestinal flora (eg antibiotics)
- Geen medicatiegebruik van geneesmiddelen die de gastrointestinale pH kunnen beïnvloeden (bijv maagzuurremmers) No medication use of drugs that may affect the gastrointestinal pH (eg, antacids)

## 3.3 3.3          Exclusiecriteria Exclusion

              Gezonde vrijwilligers Healthy volunteers

- Bekend met gastro-intestinale aandoeningen, zoals colitis ulcerosa, ziekte van Crohn, spastisch colon, colon carcinoom, ileus, stoma, maag- en/of darminfectie With known gastro-intestinal disorders such as ulcerative colitis, Crohn's disease,
   spastic colon, colon cancer, ileus, ostomy, gastric and / or intestinal infection
- Maagdarmoperatie in de hist orie (muv appendix-operatie) Gastrointestinal Operation (except appendix operation)
- Aanwezigheid *Helicobacter p ylori* , uit te sluiten met Pylobactell ® test [ 13 ] Presence *Helicobacter p ylori*

              P atiënten Patients

- Ziekte van Crohn bevindt zich in het actieve stadium Crohn's disease is in the active stage (Harvey Bradshaw ≥ 4) (Harvey Bradshaw ≥ 4)
- Aanwezigheid *Helicobacter p ylori* , uit te sluiten met Pylobactell ® test [ 13 ] Presence *Helicobacter p ylori*

## 3.4 3.4          Berekening studiegrootte Calculation study size

Deze studie is een biobeschikbaarheidstudie . This study is a bioavailability study. Calculation of studysize is not addressed. Twelve evalueerbare proefpersonen is een algemeen geaccepteerde populatiegrootte voor dit soort studies [8 , 14 , 15 ] . Rekening houdende met uitval worden evaluable subjects is a generally accepted population size for this kind of studies [8, 14, 15]. Considering some drop-out 16 proefpersonen per populatie geïncludeerd.16 subjects of each population will be included.

# 4. 4.       MEDICATIE MEDICATION

## 4.1 4.1          Toegepast geneesmiddel Applied medicine

^13^ C-ureum 50 mg modified-release tablet ^13^ C-urea 50 mg modified-release tablet

^15^ N _2_ -ureum 50 mg tablet ^15^ N _2_ -urea 50 mg tablet

## *4.2* *4.2*          *Toegepast medisch hulpmiddel* *Applied medical device*

              IntelliCap® (bijlage 1) IntelliCap® (Annex 1)

## 4.3 4.3          Samenvatting informatie uit niet-klinische studies Summary information from non-clinical studies

Zie het *Investigational Medicinal Product Dossier (IMPD).* See the *Investigational Medicinal Product Dossier (IMPD).*

## 4.4 4.4          Bijwerkingen Side effects

Zie het *Investigational Medicinal Product Dossier (IMPD).* See the *Investigational Medicinal Product Dossier (IMPD).*

## 4.5 4.5          Dosering, toedienvorm en toedienroute Dosage / Dosage form / route of administration

Zie 2.1 See 2.1

# 5. 5.       METHODE METHOD

## 5.1 5.1          Primaire uitkomstmaten Primary outcomes

- - Lokale biobeschikbaarheid bij gezonde vrijwilligers en patiënten met de ziekte van Crohn.Local bioavailability in healthy volunteers and patients with Crohn's disease. Dit is de cum PDR ^13^ C -ureum This is the cum PDR ^13^ C -urea [[1]](http://translate.googleusercontent.com/translate_f#_ftn1%23_ftn1) [[1]](http://translate.googleusercontent.com/translate_f#_ftn1%23_ftn1) gecorrigeerd voor de gemiddelde cumPDR verkregen met ^13^ C-bicarbonaat zoals gemeten in het vorige onderzoek [6] corrected for the average cumPDR obtained by ^13^ C-bicarbonate as measured in the previous study [6]
- - Tijd tussen inname en respons bij gezonde vrijwilligers en patiënten met de ziekte van Crohn.Time between intake and response in healthy volunteers and patients with Crohn's disease. Dit is de cumPDR _5%_ : het tijdstip waarop de afgifte groter is dan 5% va n de maximale cumPDR ( ^13^ C-ureum) . This is the cumPDR _5%_: at the time when release is exceeds 5%, of the maximum cumPDR ^(13^ C-urea).
- -Pulse time in healthy volunteers and patients with Crohn's disease. Dit is het verschil tussen tijdstippen waarop de maximale PDR en de PDR _5%_ This is the difference between times at which the maximum PDR is reached and the lagtime.
- -Description of the gastro-intestinal pH profile of healthy volunteers

## 5.2 5.2          Studieprocedures Study Procedures

Van de proefpersonen worden op vastgestelde tijden ademmonsters en urine monsters afgenomen. Breath samples and urine samples at fixed intervals. In totaal betreft het 23-25 ademmonsters per test en 6 urinemonsters. Van de gezonde vrijwilligers worden een pH-profiel opgenomen met behulp van de IntelliCap®. In total, the 23-25 ​​breath samples per test and six urine samples are taken. Of the healthy volunteers, a pH profile recorded with the aid of the IntelliCap®.

## 5.3 5.3          Tussentijds stoppen Withdrawal

De proefpersoon heeft te allen tijde het recht zich terug te trekken uit het onderzoek. The subject at all times have the right to withdraw from the study.

# 6. 6.       VEILIGHEIDSRAPPORTAGE Safety reporting

## 6.1 6.1          Sectie 10 WMO Section 10 WMO

In overeenstemming met sectie 10, subsectie 1 van de WMO, zal de onderzoeker de proefpersonen en de METc informeren, als uit voortschrijdend inzicht blijkt dat deelname risico's met zich meebrengt die niet waren voorzien. In accordance with section 10, subsection 1 of the WMO, the investigator will inform the subjects and the METc when unforeseen risks occur.

## 6.2 6.2          Risico's proefpersonen Risk subjects

De tabletten bevatten: The tablets contain:

- - Farmaceutische stoffen, die voor humane toepassing goedgekeurd zijn. Pharmaceutical substances, which have been approved for human
     application.
  - Ureum, een lichaamseigen stof die als marker in kleine hoeveelheden in de vorm van een stabiel, niet radioactief isotoop wordt ingenomen. Urea, an endogenous substance that as a marker in small quantities in
     the form of a stable, non-radioactive isotope is ingested.
  - Coffeïne, in een kleine (subtherapeutische) hoeveelheid, die slechts eenmalig wordt ingenomen. Caffeine in a small (sub-therapeutic) amount that is taken only once.

- Retentie van de IntelliCap kan optreden, waardoor endoscopische of chirurgische verwijdering noodzakelijk is (alleen van toepassing voor de gezonde vrijwilligers). Retention of IntelliCap can occur, making endoscopic or surgical removal (only applicable for healthy volunteers) necessary.

In the "Clinical Investigators Brochure" of the IntelliCap (version 2 .0) a review of the literature data of different devices (with similar size) which are used in various applications can be found. Hieruit kan geconcludeerd worden dat er geen capsule retentie is opgetreden bij gezonde vrijwilligers. It can be concluded that no capsule retention occurred in healthy volunteers.

De monstername (adem en urine) geeft geen risico voor de proefpersonen. Sampling (breath and urine) indicates no risk to subjects.

# 7. 7.       ANALYSE ANALYSIS

## 7.1 7.1          Analyse Analysis

De ademmonsters worden geanalyseerd op ^13^ CO _2_ . The breath samples are analyzed for ^13^ CO _2_.

De urine monsters worden geanalyseerd op ^1 5^ N _2_ , The urine samples are analyzed for ^1 5^ N _2_, ^13^ C , totale N en totale C. ^13^ C, total nitrogen, and total C.

pH-waarden worden uitgelezen vanuit de data-recorder en een 24-h pH-profiel wordt vastgelegd. pH values ​​are determined from the data recorder, and a pH profile will be made

## 7.2 7.2          Laboratoriumtechnieken Laboratory Techniques

De isotoop verrijkingen ( ^13^ CO _2_ en ^15^ N _2_ -ureum ) The isotopic enrichments ^(13^ CO _2_ and ^15^ N _2_ urea) in de adem- en urine monsters wordt gemeten met behulp van isotope-ratio-mass spectrometry (IRMS) met behulp van een gevalideerde analyse methode [ 15 , 16 ] . in the breath and urine samples will be measured with the aid of isotope-ratio mass spectrometry (IRMS) by means of a validated assay method [15, 16].

## *7.3* *7.3*          *I ntelliCap®* *IntelliCap®*

Zie bijlage 1 (“Clinical Investigator's Brochure”: informatie IntelliCap® Philips). See Appendix 1 ("Clinical Investigator's Brochure").

# 8. 8.       ETHISCHE OVERWEGINGEN ETHICAL CONSIDERATIONS

## 8.1 8.1          Vereiste verklaringen Required statements

De studie wordt uitgevoerd volgens de principes van de Declaration of *Seoul* versie 2008 en de volgens de principes van de WMO *.* Voor de daadwerkelijke start is goedkeuring door een erkende medisch-ethische toetsingscommissie nodig. The study is conducted according to the principles of the Declaration of *Seoul* 2008 version and according to the principles of the *WMO.* For the actual start is approval by an accredited ethics committee required.

## 8.2 8.2          Werving en consent Recruitment and consent

De gezonde vrijwilligers worden geworven middels een advertentie. De patiënten worden geïncludeerd via hun behandeldend arts. Healthy volunteers are recruited through an advertisement. Patients are enrolled through their treating physician. Alle proefpersonen worden schriftelijk ingelicht over het doel, de aard, de duur en de bezwaren en risico's van deelname (zie 'informatie voor proefpersonen' en 'toestemmingsverklaring voor deelname aan het wetenschappelijk onderzoek'. Indien gewenst kan de proefpersoon aanvullend informatie inwinnen bij een onafhankelijk arts. All subjects will be informed in writing of the purpose, nature, duration and the drawbacks and risks of participation (see 'Information for subjects' and' informed consent to participate in research. "If desired, the subject can additionally obtain information from an independent doctor.

## 8.3 8.3          Verzekering Insurance

AccordingIngevolge art.AccoAA to art. 7 van de Wet medisch wetenschappelijk onderzoek met mensen (Stbl. 1998, 161) is voor de deelnemende proefpersonen een verzekering afgesloten die de door het onderzoek veroorzaakte schade door dood of letsel van de deelnemende proefpersonen dekt.7 of the Medical Research Involving Human Subjects (Stbl. 1998, 161) there will be an insurance by which the participating subjects are covered. Deze verzekering voldoet aan de bepalingen van het Besluit verplichte verzekering bij medisch-wetenschappelijk onderzoek met mensen (Stbl. 2003, 266).This insurance complies with the provisions of the Decree on compulsory insurance in medical research involving human subjects (Sb., 2003, 266). Aan het onderzoek deelnemende proefpersonen zullen schriftelijk worden ingelicht over deze verzekering. Participated subjects will be informed in writing of this insurance.

## 8.4 8.4          Vergoeding Compensation

De proefpersonen wordt een financiële vergoeding geboden van in totaal 50 The subjects are offered a financial compensation of 50 euro for each testday including traveling costs.

# 9. 9.       ADMINISTRATIEVE ASPECTEN EN PUBLICATIE ADMINISTRATIVE ASPECTS AND PUBLICATION

## 9.1 9.1          Archivering studiedocumentatie Archiving study documentation

Alle gegevens van de studie zullen worden bewaard in het archief van de apotheek, gedurende een periode van tenminste 15 jaar. All data from the study will be kept in the archives of the pharmacy for a period of at least 15 years.

## 9.2 9.2          Amendementen Amendments

Relevante wijzigingen –in termen van de METc-aanvraag - in het door de METc goedgekeurde protocol worden opgenomen in een amendement en zullen ter beoordeling worden aangeboden aan de METc. Relevant changes -in terms of METc request – will be included in the approved protocol as an amendment and will be presented for review to the METc.

## 9.3 9.3          Voortgangsrapportage Progress Report

Indien het onderzoek na een jaar nog niet is afgerond zal de onderzoeker voortgangsrapportage van het onderzoek opstellen en voorleggen aan de METc. If the investigation after a year has not been completed the investigator will report the progress of the study to the METc.

## 9.4 9.4          Rapportage studieresultaten Reporting study results

De onderzoeker zal de resultaten na afronden van de studie aan de METc bekendmaken, binnen de gestelde periode van (90 dage n ). The researcher will report the results after completion of the study to the METc, within the prescribed period (90 day s). Het eind van de studie is dat moment, wanneer van de laatste patiënt de laatste monsters zijn afgenomen. The end of the study is that moment when the last patient and the last sampling are finished. Binnen een jaar na afronden van de studie zal de onderzoeker de definitieve rapportage van de studie voorleggen aan de METc. Within one year after completion of the study, the investigator will submit the final report of the study on the METc.

Wanneer de studie voortijdig wordt beëindigd zal de onderzoeker dit bekendmaken aan de METc binnen de gestelde termijn van 15 dagen, voorzien van een onderbouwing voor het afbreken van de studie. When the study is terminated early, the researcher will report this to the METc within the prescribed period of 15 days and will provide the reason for the termination of the study.

## 9.5 9.5          Openbaarmaking en publicatie Disclosure and publication

De resultaten zullen worden gepubliceerd. The results will be published.

# 10. 10.    REFERENTIES REFERENCES

1. Hommes DW, Oldenburg B et al. Hommes DW, Oldenburg B et al. Guidelines for treatment with infliximab for Crohn's disease. Neth J Med 2006;64(7):219-229. Guidelines for treatment with infliximab for Crohn's disease Neth J Med 2006; 64 (7):. 219-229.
2. D'Haens G, Baert F et al. Early combined immunosuppression or conventional management in patients with newly diagnosed Crohn's disease: an open randomised trial. Lancet 2008;371(9613):660-667. D'Haens G, Baert F et al Early combined immunosuppression or conventional management in patient's with Newly Diagnosed Crohn's disease: an open randomized trial Lancet 2008; 371 (9613).. 660-667.
3. Schellekens RC, Stuurman FE, van der Weert FH, Kosterink JG, Frijlink HW . Schellekens RC Stuurman FE, van der Weert FH Kosterink JG, Frijlink HW. A novel dissolution method relevant to intestinal release behaviour and its application in the evaluation of modified release mesalazine products . Eur J Pharm Sci 2007 ; 30(1):15-20. A novel dissolution method relevant to intestinal release behavior and application notes in the evaluation of modified release mesalamine products Eur J Pharm Sci 2007; 30 (1):. 15-20.
4. RCA Schellekens, F Stellaard, D Mitrovic, FE Stuurman, JGW Kosterink, HW Frijlink, Improved pulsatile drug delivery to the ascending colon by structured incorporation of disintegrants in pH-responsive polymer coatings: *in vitro* investigations and *in vivo* proof of concept, J. Control Rel 2008; 132:91-98. RCA Schellekens, F Stellaard, D Mitrovic FE Stuurman, JGW Kosterink HW Frijlink, Improved pulsatile drug delivery to the ascending colon by structured incorporation or disintegrants in pH-responsive polymer coatings: *in vitro* investigations and *in vivo* proof of concept, J. Riot Control 2008; 132: 91-98.
5. Schellekens RCA, Frijlink HW, Patent WO/2007/013794, pH-controlled pulsatile delivery system, methods for preparation and use thereof. Schellekens RCA Frijlink HW, Patent WO / 2007/013 794, pH-controlled pulsatile delivery system, methods for preparation and use thereof.
6. Schellekens RCA , Olsder GG , Langenberg SMCH , Boer T , Schellekens RCA Olsder GG, Langenberg SMCH, Farmer T, Woerdenbag HJ , Woerdenbag HJ, Frijlink HW , Frijlink HW, Kosterink JGW , Kosterink JGW, Stellaard F. Proof-Of-Concept s tudy o n t he s uitability o f ^13^ C - u rea as a marker substance for assessment of *in vivo* behaviour of oral colon - targeted dosage forms . Br J Pharmacology Stellaard F. Proof-Of-Concept s tudy o n o f t he s uitability ^13C^ - you rea as a marker substance for assessment or *in vivo* behavior or oral colon -. Targeted dosage forms Br J Pharmacology 2009 : 2009: accepted for publication . accepted for publication .
7. Olsde r GG , Olsde r GG Schellekens RCA , Frijlink HW , Schellekens RCA Frijlink HW Stellaard F , Kosterink JGW. Stellaard F Kosterink JGW. Local bio-availability of a n oral colon targeted dosage form: a phase I-study in healthy volunteers, in preparation. Local bio-availability of a n oral colon targeted dosage form: a Phase I study in healthy volunteers, in preparation.
8. *EMEA, NfG on the investigation of bioavailbility and bioequivalence, 2002. EMEA NfG on the investigation or bioavailbility and bioequivalence, 2002.*
9. Misaki F and Kawai K. The estimation of gastric secretory capacity by the telemering method of pH-sensitive radiocapsule. Gastroenterologia Japonica 1976;11: 100- 4 Misaki Kawai F and K. The estimation of gastric secretory capacity by the telemering method of pH-sensitive radio capsule Gastroenterologia Japonica 1976; 11:. 100- 4
10. Wang WX, Yan GZ, Sun F, Jiang PP, Zhang WQ, Zhang GF. Wang WX, GZ Yan, Sun F, Jiang PP, WQ Zhang, Zhang GF. A non-invasive methode for gastrointestinal parameter monitoring. A non-invasive method for gastrointestinal parameter monitoring. World J Gastroenterol 2005;11:521-4 World J Gastroenterol 2005; 11: 521-4
11. Biao H, Guozheng Y and Peng Z. Multi-sensor radiotelemetry system for intestinal motility measurement. Biao H, Guozheng Y and Z. Peng Multi-Sensor radiotelemetry system for intestinal motility measurement. J Med Engin Techn 2009;33:66-71 J Med Tech Engin 2009; 33: 66-71
12. Verbeke K et al. In vivo evaluation of a colonic delivery system using isotope techniques, A liment Pharmacol Ther 2005 ; 21: 1 87 - 194 . Verbeke K et al In vivo evaluation of a colonic delivery system using isotopic techniques, A liment Pharmacol Ther 2005; 21: 1 87-194..
13. 1B-tekst Pylobactell, 100 mg ^13^ C-ureum, oplosbare tablet 1B-text Pylobactell 100 mg ^13^ C-urea, soluble tablet
14. Bott C et al. In vivo evaluation of a novel pH- and time-based multiunit colonic drug delivery system, Aliment Pharmacol Ther 2004 ; 20:347 - 353. Bott et al C. In vivo evaluation of a novel pH and time-based multi-unit colonic drug delivery system, Aliment Pharmacol Ther 2004; 20: 347-353..
15. Wutzke KD, Sattinger V. Wutzke KD Sattinger V. 15N-excretion of heat-killed Lactobacillus casei in humans. 15N-excretion or heat-killed Lactobacillus casei in humans. Eur J Clin Nutr 2006 ; 60(7):847-52. Eur J Clin Nutr 2006; 60 (7): 847-52.
16. Stellaard F, Geypens B. European interlaboratory comparison of breath 13CO2 analysis. Stellaard F Geypens B. European inter-laboratory comparison of breath 13CO2 analysis. Gut 1998;43:S2-S6. Gut 1998; 43: S2-S6.


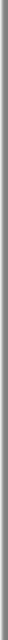


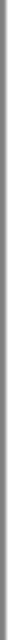


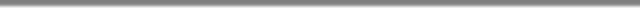


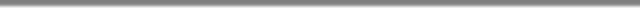


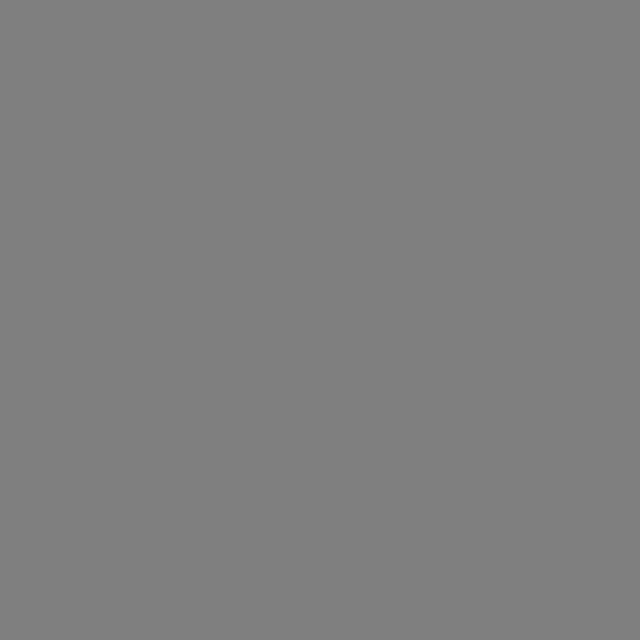


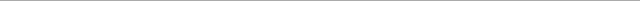


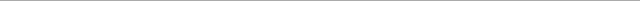


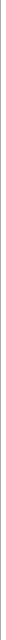


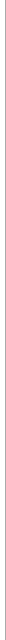


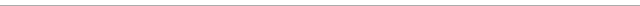


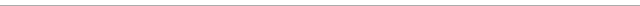


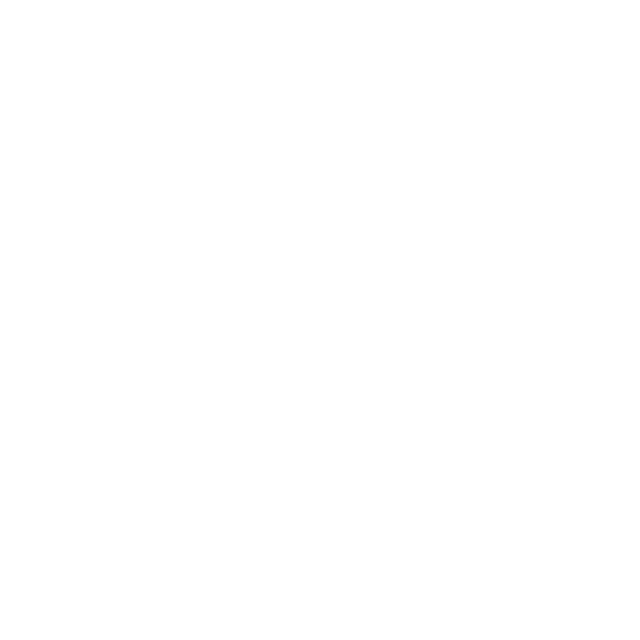


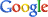


**Originele tekst in het Nederlands:**

Ten tweede, de conventionele onderhoudsbehandeling (elke 8 weken 5 mg/kg) sluit niet aan op de pathofysiol o gie van de ziekte.


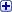
Een betere vertaling voorstellen


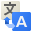


**Oorspronkelijke tekst**

Een betere vertaling bijdragen
