# Supplementary material for: Gastrointestinal pH and Transit Time Profiling in Healthy Volunteers Using the IntelliCap System Confirms Ileo-Colonic Release of ColoPulse Tablets
Source: PLoS One. 2015 Jul 15;10(7):e0129076. doi: 10.1371/journal.pone.0129076 (PMC4503763; doi:10.1371/journal.pone.0129076)
Supplement: S2 File — (DOC) [file pone.0129076.s003.doc]

**PROTOCOL BIOCOP-2-STUDIE**

**Biobeschikbaarheid van een ColoPulse®-tablet**

**PROTOCOL TITEL:**

BIOCOP-2-studie

**PROTOCOL ONDERTITEL:**

Studie naar de lokale biobeschikbaarheid uit een "modified-release" tablet (ColoPulse®-technologie) bij gezonde vrijwilligers en patiënten.

| **Korte titel** | Colon-specifieke afgifte studie (2) |
| --- | --- |
| **Versie** | 4.0 |
| **Datum** | 11-10-2010 |
| **EudraCT** | 2009-013471-21 |
| ***METc*** | 2009.188 |
| **Onderzoeksgroep** | **Drs. J.M. Maurer, ziekenhuisapotheker i.o.**  **Drs. H.M. van Rieke, ziekenhuisapotheker i.o.**  **Drs. R.C.A. Schellekens, ziekenhuisapotheker**  **Dr. J.G.W. Kosterink, ziekenhuisapotheker-klinisch-farmacoloog**  Klinische Farmacie & Apotheek  UMC Groningen  **Dr. G. Dijkstra, gastro-enteroloog**  Interne Geneeskunde  UMC Groningen  **Prof. Dr. H.W. Frijlink, farmaceutisch technoloog**  **Dr. H.J. Woerdenbag, apotheker**  Farmacie en Farmaceutische Wetenschappen  Rijksuniversiteit Groningen  **Dr. F. Stellaard, biochemicus**  Pathologie & Laboratoriumgeneeskunde  UMC Groningen |
| **Sponsor** | **UMC Groningen**  **Dr. J.G.W. Kosterink, ziekenhuisapotheker-klinisch-farmacoloog**  Hoofd Klinische Farmacie & Apotheek  UMC Groningen |
| **Onafhankelijk arts** | **Drs. R.J. Wijdh, oogarts**  Afdeling Oogheelkunde  UMC Groningen |

**PROTOCOL SIGNATURE SHEET**

| **Naam** | **Handtekening** | **Datum** |
| --- | --- | --- |
| Dr. J.G.W. Kosterink, ziekenhuisapotheker  Hoofd Klinische farmacie & Apotheek  UMC Groningen |  |  |
| Drs. R.C.A. Schellekens, ziekenhuisapotheker  Klinische farmacie & Apotheek  UMC Groningen |  |  |
| Drs. J.M. Maurer, ziekenhuisapotheker i.o.  Klinische farmacie & apotheek  UMC Groningen |  |  |
| Drs. H.M. van Rieke, ziekenhuisapotheker i.o.  Klinische farmacie & apotheek  UMC Groningen |  |  |
| Dr. G. Dijkstra, gastro-enteroloog  Interne Geneeskunde  UMC Groningen |  |  |

**INHOUDSOPGAVE**

1. INTRODUCTIE EN RATIONALE [8](#__RefHeading___Toc269396766)

2. STUDIE OPZET [9](#__RefHeading___Toc269396767)

2.1 Principe [9](#__RefHeading___Toc269396768)

2.2 Uitvoering studie [10](#__RefHeading___Toc269396769)

3. STUDIEPOPULATIE [14](#__RefHeading___Toc269396770)

3.1 Proefpersonen [14](#__RefHeading___Toc269396771)

3.2 Inclusiecriteria [14](#__RefHeading___Toc269396772)

3.3 Exclusiecriteria [14](#__RefHeading___Toc269396773)

3.4 Berekening studiegrootte [14](#__RefHeading___Toc269396774)

4. MEDICATIE [15](#__RefHeading___Toc269396775)

4.1 Toegepast geneesmiddel [15](#__RefHeading___Toc269396776)

*4.2* Toegepast medisch hulpmiddel [15](#__RefHeading___Toc269396777)

4.3 Samenvatting informatie uit niet-klinische studies [15](#__RefHeading___Toc269396778)

4.4 Bijwerkingen [15](#__RefHeading___Toc269396779)

4.5 Dosering, toedienvorm en toedienroute [15](#__RefHeading___Toc269396780)

5. METHODE [15](#__RefHeading___Toc269396781)

5.1 Primaire uitkomstmaten [15](#__RefHeading___Toc269396782)

5.2 Studieprocedures [16](#__RefHeading___Toc269396783)

5.3 Tussentijds stoppen [16](#__RefHeading___Toc269396784)

6. VEILIGHEIDSRAPPORTAGE [16](#__RefHeading___Toc269396785)

6.1 Sectie 10 WMO [16](#__RefHeading___Toc269396786)

6.2 Risico’s proefpersonen [16](#__RefHeading___Toc269396787)

7. ANALYSE [17](#__RefHeading___Toc269396788)

7.1 Analyse [17](#__RefHeading___Toc269396789)

7.2 Laboratoriumtechnieken [17](#__RefHeading___Toc269396790)

*7.3* IntelliCap® [17](#__RefHeading___Toc269396791)

8. ETHISCHE OVERWEGINGEN [18](#__RefHeading___Toc269396792)

8.1 Vereiste verklaringen [18](#__RefHeading___Toc269396793)

8.2 Werving en consent [18](#__RefHeading___Toc269396794)

8.3 Verzekering [18](#__RefHeading___Toc269396795)

8.4 Vergoeding [18](#__RefHeading___Toc269396796)

9. ADMINISTRATIEVE ASPECTEN EN PUBLICATIE [19](#__RefHeading___Toc269396797)

9.1 Archivering studiedocumentatie [19](#__RefHeading___Toc269396798)

9.2 Amendementen [19](#__RefHeading___Toc269396799)

9.3 Voortgangsrapportage [19](#__RefHeading___Toc269396800)

9.4 Rapportage studieresultaten [19](#__RefHeading___Toc269396801)

9.5 Openbaarmaking en publicatie [19](#__RefHeading___Toc269396802)

10. REFERENTIES [20](#__RefHeading___Toc269396803)

**LIJST MET AFKORTINGEN EN RELEVANTE DEFINITIES**

| **ABR** | **Algemene Beoordeling en Registratie formulier** |
| --- | --- |
| **CV** | **Curriculum Vitae** |
| **EudraCT** | **European drug regulatory affairs Clinical Trials GCP Good Clinical Practice** |
| **IC**  **IFX** | **Informed Consent**  **Infliximab** |
| **IMPD** | **Investigational Medicinal Product Dossier** |
| **METC**  **PDR**  **TNF** | **medisch ethische toetsing commissie (METC)**  **Percentage of Dosage Recovered**  **Tumor Necrose Factor** |
| **Wbp** | **Wet Bescherming Persoonsgevens** |
| **WMO** | **Wet Medisch-wetenschappelijk Onderzoek met Mensen** |

**Samenvatting**

**Rationale:**

Infliximab (IFX) als eerste TNF-alfa remmer heeft de farmacotherapeutische mogelijkheden voor patiënten met de ziekte van Crohn aanmerkelijk verbeterd [1,2]. Dit geldt zowel voor de inductie van remissie als het onderhoud daarvan. De huidige therapie kent echter een aantal nadelen [1]. Ten eerste, de verbeterde effectiviteit gaat gepaard met het optreden van ernstige bijwerkingen (ernstige infecties zoals tuberculose, hartfalen, auto-immuniteit en maligniteiten). Deze bijwerkingen zijn alle gerelateerd aan de systemische blootstelling aan IFX.

Ten tweede, de conventionele onderhoudsbehandeling (elke 8 weken 5 mg/kg) sluit niet aan op de pathofysiologie van de ziekte. Dit blijkt onder andere uit de klinische observatie dat een significant aantal patiënten zich reeds eerder dan deze 8 weken meldt voor een nieuw infuus. Dit fenomeen wordt verklaard uit het feit dat na IFX-behandeling het aantal TNF-alfa producerende cellen in de lamina propria weer langzaam toeneemt, hetgeen leidt tot een toenemende inflammatoire reactie.

Bovenstaande beide nadelen van de onderhoudsbehandeling zijn mogelijk te voorkomen met een lokale en frequentere (dagelijkse) blootstelling van de darmwand aan een lage dosis IFX. In een multi-step project wil de onderzoeksgroep deze benadering verder onderzoeken in de betreffende patiëntpopulatie. In een eerder stadium is in de BIOCOP-1 studie de lokale biobeschikbaarheid van een “*modified release*” capsule onderzocht in gezonde vrijwilligers. Het vervolg van het project bestaat uit de volgende twee delen:

1. Studie naar de lokale biobeschikbaarheid uit een "*modified-release*" tablet (ColoPulse®-technologie) bij gezonde vrijwilligers en patiënten met de ziekte van Crohn **(BIOCOP-2)**
2. Studie naar de effectiviteit en veiligheid van een "*modified-release*" tablet (ColoPulse®-technologie) bij patiënten met de ziekte van Crohn **(TOMATE-1)**.

Dit studieprotocol beschrijft het eerste deel, de zogenaamde **BIOCOP-2**-studie.

**Doel:**

Vaststellen lokale biobeschikbaarheid "modified-release" tablet (ColoPulse®-technologie) bij gezonde vrijwilligers en patiënten met de ziekte van Crohn.

Vaststellen/opnemen van gastro-intestinale pH profiel bij gezonde vrijwilligers.

**Studie-opzet:**

Prospectieve biobeschikbaarheidstudie

**Studiepopulatie**

Proefpersonen: gezonde vrijwilligers en patiënten met de ziekte van Crohn (in remissie).

**Interventie**:

Toedienen ^13^C-ureum 50 mg modified-release tablet + ^15^N_2_-ureum 50 mg immediate release tablet met twee verschillende inname-instructies en het afnemen van adem en urinemonsters tbv het vaststellen van de biobeschikbaarheid.

Inname IntelliCap tbv het vaststelen van het gastro-intestinale pH-profiel.

**Primaire uitkomstmaten:**

1. Lokale biobeschikbaarheid, uitgedrukt als de fractie ^13^C die in de vorm van ^13^CO_2_ wordt teruggevonden in de adem bij gezonde vrijwilligers en patiënten met de ziekte van Crohn.
2. Tijd tussen inname en respons bij gezonde vrijwilligers en patiënten met de ziekte van Crohn. Dit is het tijdstip waarop de afgifte groter is dan 5% van de maximale cumPDR* van ^13^C-ureum..
3. Pulse time bij gezonde vrijwilligers en patiënten met de ziekte van Crohn. Dit is het verschil tussen de tijdstippen waarop de maximale PDR en de PDR_5%_ wordt bereikt.
4. Beschrijving van het gastro-intestinale pH-profiel van gezonde vrijwilligers

**Risico’s deelname:**

De tabletten bevatten:

- Farmaceutische stoffen, die voor humane toepassing zijn goedgekeurd
- Ureum, een lichaamseigen stof die als marker in kleine hoeveelheden in de vorm van een stabiel, niet radioactief isotoop wordt ingenomen.
- Coffeïne, in een kleine (subtherapeutische) hoeveelheid, die slechts eenmalig wordt ingenomen.

Retentie van de IntelliCap kan theoretisch gezien optreden, waardoor endoscopische of chirurgische verwijdering noodzakelijk is (wordt alleen toegepast bij gezonde vrijwilligers).

In de “Clinical Investigator’s Brochure” (versie 2.0) is een literatuuroverzicht gegeven van verschillende, wat betreft afmeting gelijkende, devices, die gebruikt worden bij verschillende toepassingen. Hieruit kan geconcludeerd worden dat er geen capsule retentie is opgetreden bij gezonde vrijwilligers.

De monstername (adem en urine) geeft geen risico voor de proefpersonen.

# INTRODUCTIE EN RATIONALE

Verbeteren behandeling ziekte van Crohn

Infliximab (IFX, Remicade®) is een anti-TNF antilichaam dat is geregistreerd voor inductie- en onderhoudsbehandeling van patiënten met therapieresistente ziekte van Crohn (1 maal per 8 weken een dosis van 5 mg/kg intraveneus). De effectiviteit van IFX is aangetoond bij zowel de actieve luminale ziekte als bij enterocutane fisteling. IFX kan ook worden toegepast bij extra-intestinale verschijnselen van de ziekte van Crohn zoals pyoderma gangrenosum, uveitis en arthropathie [1]. Recent is aangetoond dat vroege behandeling met IFX mogelijk effectiever is dan de standaard step-up behandeling waarbij alleen therapieresistente patiënten dit middel krijgen toegediend [2]. Afgezien van het wegvangen van anti-TNF wordt de effectiviteit van IFX en adalimumab vooral toegeschreven aan het vermogen om geactiveerde mucosale T-cellen waarbij TNF op het celoppervlak aanwezig is tot apoptose te brengen. Na een infuus komen deze cellen echter terug waardoor een onderhoudsbehandeling en combinatietherapie met afweerremmende middelen (azathioprine, methotrexaat) noodzakelijk wordt. Een probleem van de intermitterende intraveneuze of subcutane toediening vormen de infuusreacties en loss of response. Loss of response is veelal geassocieerd met de vorming van anti-IFX antistoffen. Daarnaast is er vanwege de systemische expositie van IFX meer kans op systemische bijwerkingen waarbij infecties en in het bijzonder tuberculose het meest gevreesd worden. Om systemische expositie te vermijden, zijn er met name bij fistels studies gedaan met locale injectie van IFX. Bij de ziekte van Crohn is de darmmucosa vooral ter plaatse van de ontsteking verhoogd permeabel. De ontsteking zit meestal in het terminale ileum en colon. Mogelijk zou frequentere en/of continue lokale delivery van een anti-TNF middel ter plekke van de ontsteking de voorkeur genieten boven de 8-weekse intraveneuze toediening vanwege de lagere systemische belasting. Daarnaast zou dagelijkse lokale delivery van een lagere keerdosis mogelijk een continue apoptose-inductie kunnen veroorzaken van zich ontwikkelende T-cellen, hetgeen beter aansluit bij de pathofysiologische processen. Verder zijn er minder systemische bijwerkingen en anti-stofvorming te verwachten en hoeft patiënt geen infusen of injecties meer de ondergaan. Kortom, de benefit-risk van IFX-behandeling zou verbeteren, hetgeen bij de ziekte van Crohn een doorbraak betekent.

Ontwikkeling toedieningsvorm

In een samenwerkingsverband van UMCG en RUG is een technologie ontwikkeld waarmee na orale inname van een tablet of capsule selectieve afgifte van geneesmiddelen kan plaatsvinden in het terminale ileum / proximale colon [3,4,5]. Voordat een patiëntenstudie naar de uitkomsten van topicale behandeling met oraal IFX kan plaatsvinden, moet eerst gevalideerd worden dat het afgifteprofiel van de te gebruiken formulering juist is. De opzet van deze studie bouwt op de resultaten en ervaring opgedaan in studie CO-06-01 (BIOCOP-1): “Pilotstudie naar afgifte uit een orale, colon specifieke, afgifte formulering in gezonde vrijwilligers met behulp van stabiele isotopen”. In deze studie werd de geschiktheid van isotopisch ureum als markerstof bestudeerd [6]. Ook werd in deze studie de lokale biobeschikbaarheid van een gecoate capsule onderzocht in gezonde vrijwilligers [7]. Ten behoeve van de IFX-interventie is een tablet ontwikkeld, waarvan het afgifteprofiel gevalideerd moet zijn, zowel in gezonde vrijwilligers als in patiënten met de ziekte van Crohn [8]. Dit is het doel van de **BIOCOP-2**-studie.

Om aanvullende informatie te verkrijgen over de werking van de pH-gevoelige coating van het "modified-release" tablet, wordt eenmalig het gastro-intestinale pH-profiel van gezonde vrijwilligers bepaald met behulp van de IntelliCap®.

De IntelliCap® wordt geproduceerd door Philips (zie bijlage 1). Dit is een medical device in de vorm van een capsule (afmeting: 18mm bij 11mm), dat oraal wordt ingenomen, de pH in het maagdarmkanaal meet, en wordt uitgescheiden met de ontlasting. *De IntelliCap® wordt gevuld met fysiologisch zout (NaCl 0,9%).* Het is van belang de pH in het maagdarmkanaal te volgen, omdat de desintegratie van de “modified-release” tablet afhankelijk is van de pH. Inzicht in de pH kan eventuele non-respons op de ontwikkelde tabletten verklaren. Het meten van de pH in het maagdarmkanaal met behulp van een capsule is reeds eerder in de literatuur beschreven [9-11].

**DOELSTELLINGEN**

Vaststellen dat het afgifteprofiel van de tabletformulering voldoet bij gezonde vrijwilligers (**BIOCOP-2A**) en patiënten (**BIOCOP-2B**).

Vaststellen van het gastro-intestinale pH-profiel bij gezonde vrijwilligers (**BIOCOP-2A**).

# STUDIE OPZET

## Principe

De lokale biobeschikbaarheid van het "modified-release" tablet (ColoPulse®-technologie) [5] wordt onderzocht met behulp van ureum als marker [12]. In essentie wordt dezelfde benadering toegepast als in de **BIOCOP-1**-studie.

Een "immediate release" tablet 50 mg ^15^N_2_-ureum en een "modified-release" tablet 50 mg ^13^C-ureum worden tegelijk oraal ingenomen. De tablet 50 mg ^15^N_2_-ureum zal reeds in de maag desintegreren en de ^15^N_2_-ureum wordt snel en volledig opgenomen. Uitscheiding geschiedt voor het overgrote deel renaal via de urine. De biobeschikbaarheid van ^15^N_2_-ureum zal worden gebruikt als referentie om de hoeveelheid opgenomen ^13^C uit ^13^C-ureum te bepalen.

De tablet 50 mg ^13^C-ureum zal niet desintegreren in de maag en de dunne darm vanwege de aangebrachte coating (ColoPulse®-technologie). In het terminale ileum desintegreert de tablet op basis van de lokale zuurgraad in dat darmsegment. De ^13^C-ureum komt in het terminale ileum en colon beschikbaar en wordt door het bacteriële urease gehydrolyseerd tot ^13^CO_2_ en NH_3_.

H_2_N-^13^C-NH_2_ + H_2_O → 2 NH_3_ + ^13^CO_2_

Het vrijgekomen ^13^CO_2_ wordt geabsorbeerd in de circulatie en wordt als ^13^C-bicarbonaat naar de longen vervoerd, waar het dan in de vorm van ^13^CO_2_ vrijkomt in de uitgeademde lucht.

Bij opening van de tablet in een eerder stadium (dunne darm) wordt ^13^C-ureum snel en nagenoeg volledig opgenomen in de circulatie. Het wordt dan op een zelfde wijze als ^15^N2-ureum verdeeld en renaal geëlimineerd en uitgescheiden via de urine. Door in adem ^13^CO_2_ en in urine ^15^N en ^13^C te kwantificeren kan de lokale biobeschikbaarheid van het "modified-release" tablet (ColoPulse®-technologie) berekend worden naar analogie van berekeningen zoals die gebruikt werden in de **BIOCOP-1**-studie.

Van de gezonde vrijwilligers worden een pH-profiel opgenomen met behulp van de IntelliCap®. Daarvoor draagt de gezonde vrijwilliger een data-recorder (kastje) om z’n middel, dat de pH-waarden registreert die de IntelliCap® uitzendt. De IntelliCap® meet de pH iedere 10 seconden. Het kastje met gegenereerde pH-waarden wisselt de data ook iedere 10 seconden uit met het software programma op de computer. Meer informatie hierover is te vinden in bijlage 1.

## Uitvoering studie

De studieopzet voor **BIOCOP-2A** en **BIOCOP-2B** is gelijk. De proefpersonen zullen op 2 verschillende dagen een test ondergaan met tussen beide dagen een wash-out periode van minimaal 7 dagen, De testdagen worden testdag 1 en testdag 2 genoemd in dit protocol*.* Op testdag 1 wordt na 1 uur een zelf-gekozen ontbijt genuttigd. Op testdag 2 wordt na 3 uur een gestandaardiseerd ontbijt genuttigd. Hiermee kan een beeld worden gekregen van de mate waarin tijdstip en vorm van ontbijt een rol spelen bij de biologische beschikbaarheid uit de modified-release tablet. Deze gegevens zijn noodzakelijk voor vervolgonderzoek. Op testdag 2 nemen de gezonde vrijwilligers (BIOCOP-2A) tevens de IntelliCap® in.

Op beide testdagen worden twee tabletten tegelijk ingenomen op de testlocatie:

- 1 "modified-release" tablet 50 mg ^13^C-ureum
- 1 "immediate release" tablet 50 mg ^15^N_2_-ureum

De volgende toedieninstructie zal worden gegeven: neem beide tabletten in om 8.00 uur 's ochtends met 150 mL appelsap. Op testdag 1 wordt na 1 uur een zelf-gekozen ontbijt genuttigd. Op testdag 2 wordt na 3 uur een gestandaardiseerd ontbijt genuttigd. Naast de in tabel 1 en 2 omschreven hoeveelheden vocht (water, appelsap of thee zonder suiker en melk) en tijdstippen van de maaltijd is nuttigen van voeding niet aan instructies gebonden. Proefpersonen kunnen naar eigen voorkeur extra vocht innemen in de vorm van water, appelsap of thee zonder suiker en melk. Na inname van de tabletten worden volgens een vastgesteld schema ademmonsters en urinemonsters afgenomen (zie tabel 1 en 2).

Op de tweede testdag wordt door de gezonde vrijwilligers eenmalig een capsule ingenomen die de pH kan meten in het maagdarmkanaal (IntelliCap®) (**BIOCOP-2A**). Inname van de IntelliCap® (Philips) gebeurt gelijktijdig met de andere tabletten.

Samengevat komt dit neer op het volgende

- De proefpersonen mogen vanaf 20.00 uur ’s avonds tot de ochtend voor de test geen vast voedsel meer innemen. Drinken van water, appelsap of thee zonder suiker en melk is wel toegestaan.
- Op de testdag nemen de proefpersonen de tabletten in op de testlocatie en wordt gestart met de monstername van adem en urine.
- Op de tweede testdag nemen de gezonde vrijwilligers de IntelliCAP®, gelijktijdig met de tabletten, in op de testlocatie. Daarbij dragen zij om hun middel een kastje (data-recorder) dat de pH-waarden opvangt. De gezonde vrijwilligers nemen het kastje mee naar huis; hiermee kan gewoon bewogen worden.
- Rond 17.00 mag de proefpersoon naar huis en kan een zelfgekozen diner worden genuttigd. De hele avond worden nog adem- en urinemonsters verzameld tot het slapen en wordt geen vast voedsel ingenomen. Drinken van water, appelsap of thee zonder suiker en melk is wel toegestaan.
- De dag na de test wordt om 8.00 uur nog een laatste adem- en urinemonster verzameld. Hierna kan gewoon worden ontbeten.
- *De IntelliCap® dient te worden opgevangen uit de ontlasting en te worden ingeleverd in een opvangcontainer bij de onderzoekers.*
- De data-recorder wordt ingeleverd in overleg met de onderzoekers.

**Tabel 1: schema voor monstername en maaltijd inname testdag 1 (inname 1 uur voor ontbijt)**

| **Dag** | **Tijd** | **Actie** | **Test 1: Ademmonster**  **(Testlocatie)** | **Test 1:**  **Ademmonster**  **(Thuis)** | **Test 1:**  **Urinemonster**  **(Thuis)** | **Test 1:**  **Urinemonster**  **(Testlocatie*)*** | **Vocht** |
| --- | --- | --- | --- | --- | --- | --- | --- |
| 0 | 20.00 | Vasten |  |  |  |  |  |
| 1 | 8.00 | Monstername | X |  |  | X  (na opstaan) |  |
|  | 8.00 | Tabletten innemen |  |  |  | X | 150 mL |
|  | 9.00 | Monstername | X |  |  |  |  |
|  | 9.00 | Ontbijt |  |  |  |  | 150 ml |
|  | 9.30 | Monstername | X |  |  |  |  |
|  | 10.00 | Monstername | X |  |  |  |  |
|  | 10.30 | Monstername | X |  |  |  | 150 ml |
|  | 11.00 | Monstername | X |  |  |  |  |
|  | 11.30 | Monstername | X |  |  |  |  |
|  | 12.00 | Monstername | X |  |  |  |  |
|  | 12.30 | Monstername | X |  |  | X |  |
|  | 13.00 | Monstername | X |  |  |  |  |
|  | 13.00 | Lunch |  |  |  |  | 150 ml |
|  | 13:30 | Monstername | X |  |  |  |  |
|  | 14.00 | Monstername | X |  |  |  | 150 ml |
|  | 14.30 | Monstername | X |  |  |  |  |
|  | 15.00 | Monstername | X |  |  | X |  |
|  | 15.30 | Monstername | X |  |  |  | 150 ml |
|  | 16.00 | Monstername | X |  |  |  |  |
|  | 16.30 | Monstername | X |  |  |  | 150 ml |
|  | 17.00 | Monstername | X |  |  |  |  |
|  | 17.30 | Monstername |  | X | X |  |  |
|  | 18.00 | Monstername |  | X |  |  |  |
|  | 18.30 | Diner (rond dit tijdstip) |  |  |  |  |  |
|  | 18.30 | Monstername |  | X |  |  |  |
|  | 19.00 | Monstername |  | X |  |  |  |
|  | 19.30 | Monstername |  | X |  |  |  |
|  | 20.00 | Monstername |  | X | X |  |  |
|  | 21.00 | Monstername |  | X |  |  |  |
|  | 22.00 | Monstername |  | X |  |  |  |
|  | 23.00 | Monstername |  | X |  |  |  |
| *2* | 8.00 | Monstername |  | X | X |  |  |
|  | 8.05 | Ontbijt |  |  |  |  |  |

**Tabel 2: schema voor monstername en maaltijd inname testdag 2 (inname 3 uur voor ontbijt)**

| **Dag** | **Tijd** | **Actie** | **Test 2: Ademmonster**  **(Testlocatie)** | **Test 2:**  ***Ademmonster***  **(Thuis)** | **Test 2:**  **Urinemonster**  **(Thuis)** | **Test 2:**  **Urinemonster**  **(Testlocatie)** | **Vocht** |
| --- | --- | --- | --- | --- | --- | --- | --- |
| 0 | 20.00 | Vasten |  |  |  |  |  |
| 1 | 8.00 | Monstername | X |  |  | X |  |
|  | 8.00 | Tabletten en indien van toepassing IntelliCap® innemen |  |  |  | X | 150 mL |
|  | 9.00 | Monstername | X |  |  |  |  |
|  | 10.00 | Monstername | X |  |  |  |  |
|  | 11.00 | Ontbijt |  |  |  |  | 150 ml |
|  | 11.30 | Monstername | X |  |  |  |  |
|  | 12.00 | Monstername | X |  |  |  |  |
|  | 12.30 | Monstername | X |  |  | X | 150 ml |
|  | 13.00 | Monstername | X |  |  |  |  |
|  | 13.30 | Monstername | X |  |  |  |  |
|  | 14.00 | Lunch |  |  |  |  | 150 ml |
|  | 14.30 | Monstername | X |  |  |  |  |
|  | 15.00 | Monstername | X |  |  | X |  |
|  | 15.30 | Monstername | X |  |  |  | 150 ml |
|  | 16.00 | Monstername | X |  |  |  |  |
|  | 16.30 | Monstername | X |  |  |  | 150 ml |
|  | 17.00 | Monstername | X |  |  |  |  |
|  | 17.30 | Monstername |  | X | X |  |  |
|  | 18.00 | Monstername |  | X |  |  |  |
|  | 18.30 | Diner (rond dit tijdstip) |  |  |  |  |  |
|  | 18.30 | Monstername |  | X |  |  |  |
|  | 19.00 | Monstername |  | X |  |  |  |
|  | 19.30 | Monstername |  | X |  |  |  |
|  | 20.00 | Monstername |  | X | X |  |  |
|  | 21.00 | Monstername |  | X |  |  |  |
|  | 22.00 | Monstername |  | X |  |  |  |
|  | 23.00 | Monstername |  | X |  |  |  |
| *2* | 8.00 | Monstername |  | X | X |  |  |
|  | 8.05 | Ontbijt |  |  |  |  |  |

# STUDIEPOPULATIE

## Proefpersonen

De studie wordt uitgevoerd met 16 gezonde vrijwilligers in **BIOCOP-2A**.

De studie wordt uitgevoerd met 16 patiënten in **BIOCOP-2B**.

## Inclusiecriteria

Gezonde vrijwilligers

- Wilsbekwame, gezonde volwassenen (18-65 jaar)
- Geen medicatiegebruik gedurende de laatste 3 maanden van geneesmiddelen die de gastrointestinale flora kunnen beïnvloeden (bijv. antibiotica)
- Geen medicatiegebruik gedurende de laatste 4 weken van geneesmiddelen die de gastrointestinale passagetijd kan beïnvloeden (bijv. laxantia, maagzuurremmers, darmtonusbeïnvloedende middelen)
- Geen gebruik van NSAID’s gedurende de laatste 4 weken

Patiënten

- Wilsbekwame, volwassenen met de ziekte van Crohn (in remissie Harvey Bradshaw Index ≤3) (18-65 jaar)
- Geen medicatiegebruik gedurende de laatste 3 maanden van geneesmiddelen die de gastrointestinale flora kunnen beïnvloeden (bijv antibiotica)
- Geen medicatiegebruik van geneesmiddelen die de gastrointestinale pH kunnen beïnvloeden (bijv maagzuurremmers)

## Exclusiecriteria

Gezonde vrijwilligers

- Bekend met gastro-intestinale aandoeningen, zoals colitis ulcerosa, ziekte van Crohn, spastisch colon, colon carcinoom, ileus, stoma, maag- en/of darminfectie
- Maagdarmoperatie in de historie (m.u.v. appendix-operatie)
- Aanwezigheid *Helicobacter pylori*, uit te sluiten met Pylobactell® test [13]

Patiënten

- Ziekte van Crohn bevindt zich in het actieve stadium (Harvey Bradshaw ≥ 4)
- Aanwezigheid *Helicobacter pylori*, uit te sluiten met Pylobactell® test [13]

## Berekening studiegrootte

Deze studie is een biobeschikbaarheidstudie. Berekening van de studiegrootte is niet aan de orde. Twaalf evalueerbare proefpersonen is een algemeen geaccepteerde populatiegrootte voor dit soort studies [8,14,15]. Rekening houdende met uitval worden 16 proefpersonen per populatie geïncludeerd.

# MEDICATIE

## Toegepast geneesmiddel

^13^C-ureum 50 mg modified-release tablet

^15^N_2_-ureum 50 mg tablet

## *Toegepast medisch hulpmiddel*

IntelliCap® (bijlage 1)

## Samenvatting informatie uit niet-klinische studies

Zie het *Investigational Medicinal Product Dossier (IMPD).*

## Bijwerkingen

Zie het *Investigational Medicinal Product Dossier (IMPD).*

## Dosering, toedienvorm en toedienroute

Zie 2.1

# METHODE

## Primaire uitkomstmaten

- Lokale biobeschikbaarheid bij gezonde vrijwilligers en patiënten met de ziekte van Crohn. Dit is de cumPDR ^13^C-ureum[[1]](#footnote-2) gecorrigeerd voor de gemiddelde cumPDR verkregen met ^13^C-bicarbonaat zoals gemeten in het vorige onderzoek [6]
- Tijd tussen inname en respons bij gezonde vrijwilligers en patiënten met de ziekte van Crohn. Dit is de cumPDR_5%_: het tijdstip waarop de afgifte groter is dan 5% van de maximale cumPDR (^13^C-ureum).
- Pulse time bij gezonde vrijwilligers en patiënten met de ziekte van Crohn. Dit is het verschil tussen tijdstippen waarop de maximale PDR en de PDR_5%_ wordt bereikt.
- Beschrijving van het gastro-intestinale pH –profiel van gezonde vrijwilligers

## Studieprocedures

Van de proefpersonen worden op vastgestelde tijden ademmonsters en urinemonsters afgenomen. In totaal betreft het 23-25 ademmonsters per test en 6 urinemonsters. Van de gezonde vrijwilligers worden een pH-profiel opgenomen met behulp van de IntelliCap®.

## Tussentijds stoppen

De proefpersoon heeft te allen tijde het recht zich terug te trekken uit het onderzoek.

# VEILIGHEIDSRAPPORTAGE

## Sectie 10 WMO

In overeenstemming met sectie 10, subsectie 1 van de WMO, zal de onderzoeker de proefpersonen en de METc informeren, als uit voortschrijdend inzicht blijkt dat deelname risico’s met zich meebrengt die niet waren voorzien.

## Risico’s proefpersonen

De tabletten bevatten:

- - Farmaceutische stoffen, die voor humane toepassing goedgekeurd zijn.
  - Ureum, een lichaamseigen stof die als marker in kleine hoeveelheden in de vorm van een stabiel, niet radioactief isotoop wordt ingenomen.
  - Coffeïne, in een kleine (subtherapeutische) hoeveelheid, die slechts eenmalig wordt ingenomen.
- Retentie van de IntelliCap kan optreden, waardoor endoscopische of chirurgische verwijdering noodzakelijk is (alleen van toepassing voor de gezonde vrijwilligers).

In de “Clinical Investigator’s Brochure” (versie 2.0) is een literatuuroverzicht gegeven van verschillende, wat betreft afmeting gelijkende, devices, die gebruikt worden bij verschillende toepassingen. Hieruit kan geconcludeerd worden dat er geen capsule retentie is opgetreden bij gezonde vrijwilligers.

De monstername (adem en urine) geeft geen risico voor de proefpersonen.

# ANALYSE

## Analyse

De ademmonsters worden geanalyseerd op ^13^CO_2_.

De urinemonsters worden geanalyseerd op ^15^N_2_, ^13^C, totale N en totale C.

pH-waarden worden uitgelezen vanuit de data-recorder en een 24-h pH-profiel wordt vastgelegd.

*Controle retentie door IntelliCap® uit de ontlasting op te vangen.*

## Laboratoriumtechnieken

De isotoopverrijkingen (^13^CO_2_ en ^15^N_2_-ureum) in de adem- en urinemonsters wordt gemeten met behulp van isotope-ratio-mass spectrometry (IRMS) met behulp van een gevalideerde analysemethode [15,16].

## *IntelliCap®*

Zie bijlage 1 (“Clinical Investigator’s Brochure”: informatie IntelliCap® Philips).

# ETHISCHE OVERWEGINGEN

## Vereiste verklaringen

De studie wordt uitgevoerd volgens de principes van de Declaration of *Seoul* versie 2008 en de volgens de principes van de WMO*.*Voor de daadwerkelijke start is goedkeuring door een erkende medisch-ethische toetsingscommissie nodig.

## Werving en consent

De gezonde vrijwilligers worden geworven middels een advertentie. De patiënten worden geïncludeerd via hun behandeldend arts. Alle proefpersonen worden schriftelijk ingelicht over het doel, de aard, de duur en de bezwaren en risico’s van deelname (zie ‘informatie voor proefpersonen’ en ‘toestemmingsverklaring voor deelname aan het wetenschappelijk onderzoek’. Indien gewenst kan de proefpersoon aanvullend informatie inwinnen bij een onafhankelijk arts.

## Verzekering

Ingevolge art. 7 van de Wet medisch wetenschappelijk onderzoek met mensen (Stbl. 1998, 161) is voor de deelnemende proefpersonen een verzekering afgesloten die de door het onderzoek veroorzaakte schade door dood of letsel van de deelnemende proefpersonen dekt. Deze verzekering voldoet aan de bepalingen van het Besluit verplichte verzekering bij medisch-wetenschappelijk onderzoek met mensen (Stbl. 2003, 266). Aan het onderzoek deelnemende proefpersonen zullen schriftelijk worden ingelicht over deze verzekering.

## Vergoeding

De proefpersonen wordt een financiële vergoeding geboden van in totaal 50 euro per test inclusief reiskosten.

# ADMINISTRATIEVE ASPECTEN EN PUBLICATIE

## Archivering studiedocumentatie

Alle gegevens van de studie zullen worden bewaard in het archief van de apotheek, gedurende een periode van tenminste 15 jaar.

## Amendementen

Relevante wijzigingen –in termen van de METc-aanvraag - in het door de METc goedgekeurde protocol worden opgenomen in een amendement en zullen ter beoordeling worden aangeboden aan de METc.

## Voortgangsrapportage

Indien het onderzoek na een jaar nog niet is afgerond zal de onderzoeker voortgangsrapportage van het onderzoek opstellen en voorleggen aan de METc.

## Rapportage studieresultaten

De onderzoeker zal de resultaten na afronden van de studie aan de METc bekendmaken, binnen de gestelde periode van (90 dagen). Het eind van de studie is dat moment, wanneer van de laatste patiënt de laatste monsters zijn afgenomen. Binnen een jaar na afronden van de studie zal de onderzoeker de definitieve rapportage van de studie voorleggen aan de METc.

Wanneer de studie voortijdig wordt beëindigd zal de onderzoeker dit bekendmaken aan de METc binnen de gestelde termijn van 15 dagen, voorzien van een onderbouwing voor het afbreken van de studie.

## Openbaarmaking en publicatie

De resultaten zullen worden gepubliceerd.

# REFERENTIES

1. Hommes DW, Oldenburg B et al. Guidelines for treatment with infliximab for Crohn's disease. Neth J Med 2006;64(7):219-229.
2. D'Haens G, Baert F et al. Early combined immunosuppression or conventional management in patients with newly diagnosed Crohn's disease: an open randomised trial. Lancet 2008;371(9613):660-667.
3. Schellekens RC, Stuurman FE, van der Weert FH, Kosterink JG, Frijlink HW. A novel dissolution method relevant to intestinal release behaviour and its application in the evaluation of modified release mesalazine products. Eur J Pharm Sci 2007;30(1):15-20.
4. RCA Schellekens, F Stellaard, D Mitrovic, FE Stuurman, JGW Kosterink, HW Frijlink, Improved pulsatile drug delivery to the ascending colon by structured incorporation of disintegrants in pH-responsive polymer coatings: *in vitro* investigations and *in vivo* proof of concept, J. Control Rel 2008;132:91-98.
5. Schellekens RCA, Frijlink HW, Patent WO/2007/013794, pH-controlled pulsatile delivery system, methods for preparation and use thereof.
6. Schellekens RCA, Olsder GG, Langenberg SMCH, Boer T, Woerdenbag HJ, Frijlink HW, Kosterink JGW, Stellaard F. Proof-Of-Concept study on the suitability of ^13^C-urea as a marker substance for assessment of *in vivo* behaviour of oral colon-targeted dosage forms. Br J Pharmacology 2009: accepted for publication.
7. Olsder GG, Schellekens RCA, Frijlink HW, Stellaard F, Kosterink JGW. Local bio-availability of an oral colon targeted dosage form: a phase I-study in healthy volunteers, in preparation.
8. EMEA, NfG on the investigation of bioavailbility and bioequivalence, 2002.
9. Misaki F and Kawai K. The estimation of gastric secretory capacity by the telemering method of pH-sensitive radiocapsule. Gastroenterologia Japonica 1976;11: 100- 4
10. Wang W-X, Yan G-Z, Sun F, Jiang P-P, Zhang W-Q, Zhang G-F. A non-invasive methode for gastrointestinal parameter monitoring. World J Gastroenterol 2005;11:521-4
11. Biao H, Guozheng Y and Peng Z. Multi-sensor radiotelemetry system for intestinal motility measurement. J Med Engin Techn 2009;33:66-71
12. Verbeke K et al. In vivo evaluation of a colonic delivery system using isotope techniques, Aliment Pharmacol Ther 2005;21:187-194.
13. 1B-tekst Pylobactell, 100 mg ^13^C-ureum, oplosbare tablet
14. Bott C et al. In vivo evaluation of a novel pH- and time-based multiunit colonic drug delivery system, Aliment Pharmacol Ther 2004;20:347-353.
15. Wutzke KD, Sattinger V. 15N-excretion of heat-killed Lactobacillus casei in humans. Eur J Clin Nutr 2006;60(7):847-52.
16. Stellaard F, Geypens B. European interlaboratory comparison of breath 13CO2 analysis. Gut 1998;43:S2-S6.

1. cumPDR = cumulatieve waarde van de Percentage of the Dose Recovered [6]. [↑](#footnote-ref-2)
